# Effects of pre-competition conditioning strategies on acute sport performance in high-level athletes: a systematic review and network meta-analysis

**DOI:** 10.3389/fphys.2026.1930250

**Published:** 2026-10-01

**Authors:** Xueliang Chen, Haixu Hu

**Affiliations:** School of Sport Training, Nanjing Sport Institute, Nanjing, Jiangsu, China

**Keywords:** high-level athletes, ischemic preconditioning, post-activation performance enhancement, pre-competition conditioning, sport performance

## Abstract

**Introduction:**

This systematic review and network meta-analysis compared the effects of different pre-competition conditioning strategies on sport performance in high-level athletes and aimed to provide evidence-based guidance for optimizing competitive readiness.

**Methods:**

PubMed, Web of Science, SPORTDiscus, China National Knowledge Infrastructure, Scopus, and Embase were searched from inception until 4 August 2026 to identify randomized controlled or crossover trials examining acute pre-competition conditioning strategies in high-level athletes. Frequentist random-effects network meta-analyses used standardized mean differences (SMDs) with 95% confidence intervals (CIs). Intervention rankings were evaluated using the surface under the cumulative ranking curve (SUCRA). Sensitivity analyses used assumed within-participant correlation coefficients of 0.30, 0.50, and 0.70.

**Results:**

Eighty-nine publications contributing 90 independent datasets involving 1,609 high-level athletes were included. Four outcome-specific networks were constructed for explosive/jump, speed/change-of-direction, sport-specific, and endurance performance. No significant inconsistency was detected across networks. For explosive/jump performance, post-activation performance enhancement (PAPE) was superior to regular warm-up (WU) [SMD = 0.40, 95% CI 0.20–0.59] and ranked highest by SUCRA (91.5%). For speed/change-of-direction performance, foam rolling showed the largest effect versus WU [SMD = 0.66, 95% CI 0.10–1.21], followed in the rankings by dynamic stretching and PAPE. For sport-specific performance, ischemic preconditioning (IPC) was superior to sham stimulation [SMD = 0.54, 95% CI 0.16–0.92]. For endurance performance, remote ischemic preconditioning (RIPC) was superior to WU [SMD = 0.62, 95% CI 0.20–1.05] and ranked highest (95.1%). Sensitivity analyses did not materially alter the findings.

**Conclusions:**

Acute pre-competition conditioning strategies exert outcome-specific effects rather than universal performance benefits in high-level athletes. PAPE may be most applicable to explosive performance; foam rolling, dynamic stretching, and PAPE to speed/change-of-direction tasks; IPC to sport-specific tasks; and RIPC to endurance performance. Confidence in Network Meta-Analysis (CINeMA) ratings ranged from high to very low across comparisons; therefore, SUCRA rankings should be interpreted alongside the certainty and amount of supporting evidence. Further high-quality randomized crossover studies are needed.

**Systematic review registration:**

https://www.crd.york.ac.uk/PROSPERO/view/CRD420261368915, identifier CRD420261368915.

## 1 Introduction

High-level athletic performance is underpinned by systematic long-term training organization, planned manipulation of training load, and the accumulation of physiological, technical, and psychological adaptations. However, successful competition performance depends not only on accumulated preparedness, but also on the ability to express this preparedness at the required moment. Therefore, strategies implemented immediately before competition have received increasing attention as a practical approach to optimizing competitive readiness. These acute pre-competition conditioning strategies can be considered a refined extension of warm-up procedures and may help athletes optimize neuromuscular activation, muscle temperature, joint range of motion, and sport-specific performance capacity immediately before testing or competition (Kilduff et al., 2013; Xie et al., 2018).

Acute sport performance enhancement has been attempted through numerous pre-competition conditioning approaches, such as (i) regular warm-up (WU), (ii) static stretching (SS) and dynamic stretching (DS), (iii) foam rolling (FR), (iv) post-activation performance enhancement (PAPE), (v) ischemic preconditioning (IPC) and remote ischemic preconditioning (RIPC), and (vi) passive heat maintenance (Kilduff et al., 2013; Wilson et al., 2013; Salvador et al., 2016; Hendricks et al., 2020; Cowper et al., 2022; Li et al., 2023). Stretching-based strategies are commonly used in warm-up routines, with dynamic stretching generally showing more favorable effects than prolonged static stretching for explosive or sprint-related tasks (Li et al., 2023). Foam rolling has also been increasingly adopted as a pre-exercise strategy, although its direct performance-enhancing effect remains inconsistent (Hendricks et al., 2020). Post-activation potentiation (PAP) denotes an acute increase in involuntary twitch force, whereas post-activation performance enhancement refers to improved voluntary performance following a brief high-intensity conditioning activity. IPC and RIPC have been proposed as strategies to modulate metabolic and vascular responses before exercise (Wilson et al., 2013; Salvador et al., 2016). In addition, passive heat maintenance may help preserve the benefits of active warm-up during waiting periods between warm-up and performance (Cowper et al., 2022).

Despite the wide range of acute pre-competition conditioning strategies, the existing evidence remains difficult to translate into practical decision-making. First, previous studies and reviews have often focused on a single strategy or on direct pairwise comparisons between a limited number of interventions. As a result, the relative effectiveness of multiple pre-competition strategies cannot be adequately compared within a single analytical framework. Second, substantial differences in sport type, athlete training status, intervention parameters, recovery interval, warm-up condition, and performance test have led to inconsistent findings. Third, sport performance is multidimensional, and explosive/jump performance, speed/change-of-direction performance, sport-specific performance, and endurance performance may respond differently to the same pre-competition conditioning strategy. Therefore, conclusions based on a single outcome or a limited number of direct comparisons may not provide sufficient evidence for athletes, coaches, and practitioners.

A network meta-analysis (NMA), rather than conventional pairwise meta-analysis alone, can address these limitations by integrating both direct and indirect evidence across multiple interventions. This approach does not require all included studies to share the same comparator and allows different acute pre-competition conditioning strategies to be compared simultaneously within one evidence network. In addition, NMA enables the relative ranking of interventions according to their probability of producing favorable performance effects, which may provide more informative evidence for applied pre-competition decision-making.

Against this background, the present systematic review and NMA aimed to examine and compare the effects of different acute pre-competition conditioning strategies on sport performance in high-level athletes through direct and indirect network-analytical comparisons. Outcomes were classified into four categories according to performance characteristics: explosive/jump performance, speed/change-of-direction performance, sport-specific performance, and endurance performance. The overall findings may help athletes, coaches, and practitioners select evidence-based and outcome-specific strategies for optimizing competitive readiness immediately before competition.

## 2 Methods

### 2.1 Search and screening procedures

This systematic review and network meta-analysis was conducted in accordance with the Preferred Reporting Items for Systematic Reviews and Meta-Analyses extension for Network Meta-Analyses (PRISMA-NMA) guidelines (Hutton et al., 2015). The review protocol was registered in PROSPERO (registration number: CRD420261368915). The study protocol and methodological framework are presented in **Figure 1**.

**Figure 1:**
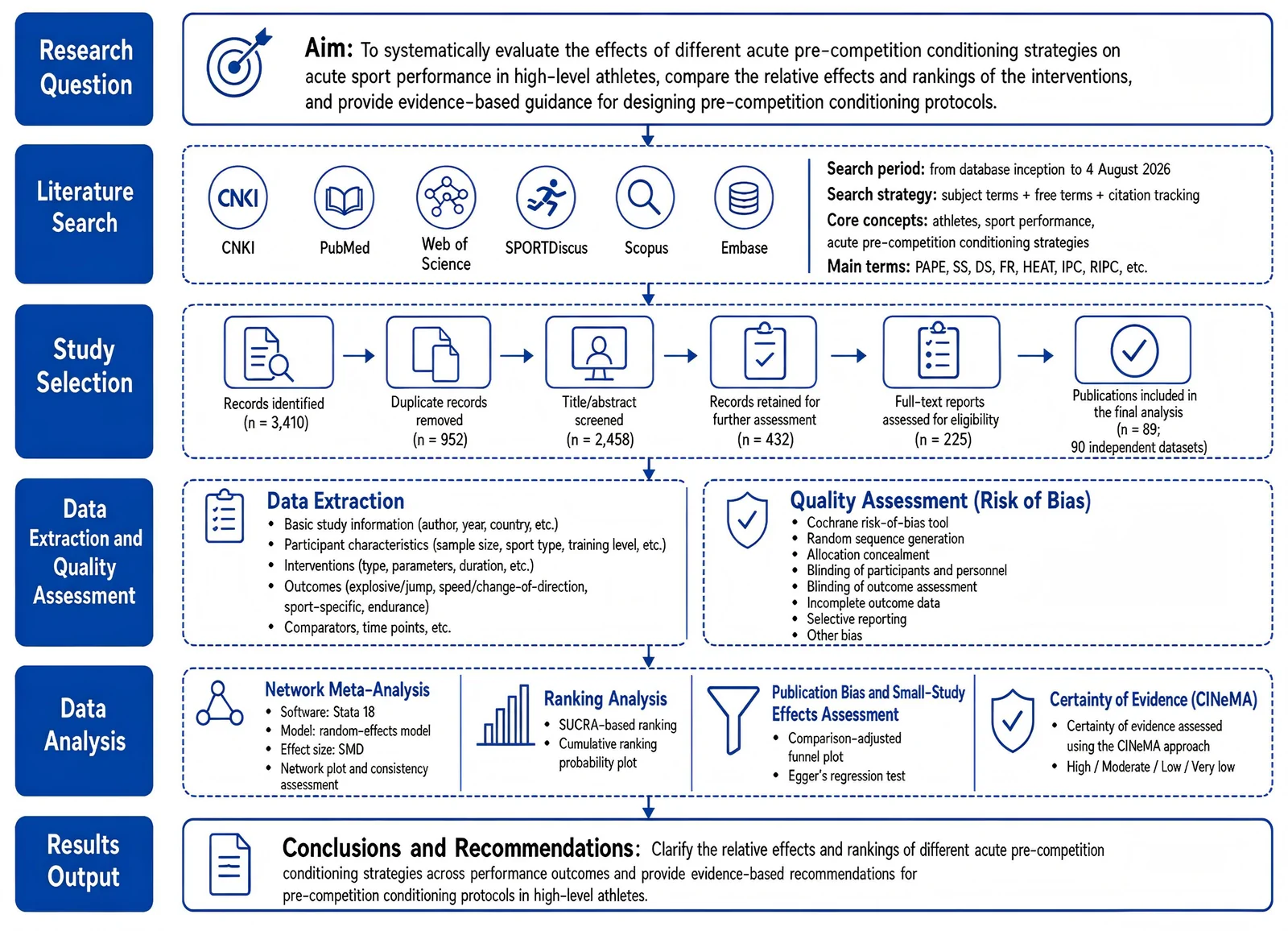
Study protocol and methodological framework.

The literature search and screening processes were independently conducted by two authors. PubMed, Web of Science, SPORTDiscus, China National Knowledge Infrastructure (CNKI), Scopus, and Embase were searched from inception to 4 August 2026. Relevant search terms were combined using Boolean operators (OR/AND) and applied to search levels covering athletes, sport performance, and acute pre-competition conditioning strategies. The intervention-related terms included post-activation performance enhancement (PAPE), post-activation potentiation, static stretching, dynamic stretching, combined stretching, foam rolling, self-myofascial release, passive heat maintenance, ischemic preconditioning (IPC), and remote ischemic preconditioning (RIPC). Searches in CNKI were mainly performed using the subject field and title/keyword/abstract field; in Web of Science, the topic field was used; in SPORTDiscus, the XB field was used; and in PubMed, Medical Subject Headings terms were combined with Title/Abstract fields; in Scopus, the TITLE-ABS-KEY field was used; and in Embase, Emtree terms were combined with title, abstract, and keyword fields. Search filters for randomized controlled trials and randomized crossover trials were applied where appropriate. In addition, citation tracking and manual searches of relevant primary articles and reviews were conducted to improve the completeness of the search. The full database-specific search strategies are provided in the Electronic **Supplementary Material**.

All retrieved records were imported into EndNote 21, and duplicates were removed using a combination of automatic and manual procedures. The remaining records were gradually screened by two authors using titles, abstracts, and full texts for potentially eligible studies. Any disagreement regarding study inclusion or exclusion was resolved through discussion with an independent third reviewer. The final decision regarding eligibility was made according to the PICOS framework [population (P), intervention (I), comparators (C), outcomes (O), and study design (S)]. The following inclusion criteria were applied: participants were healthy high-level athletes with a clearly defined sport-specific training background, including competitive, well-trained, national-level, elite, professional, or youth competitive athletes (P); interventions were acute pre-competition conditioning strategies implemented before formal testing, including PAPE, static stretching, dynamic stretching, combined stretching protocols, foam rolling, passive heat maintenance, IPC, and RIPC (I); comparators included regular warm-up, sham stimulation, passive or active control conditions, or other eligible acute pre-competition conditioning strategies (C); outcomes included at least one quantitative sport performance measure, such as jump performance, sprint performance, change-of-direction performance, sport-specific performance, power output, time to exhaustion, running economy, or time-trial performance (O); and studies were original randomized controlled trials or randomized crossover trials (S).

Studies were excluded if they involved general college students, recreationally active individuals, untrained participants, or athletes with disease or injury. Studies involving chronic training interventions, periodized training programs, nutritional supplementation alone, altitude exposure, blood flow restriction, interventions performed during or after exercise, or other non-target interventions were also excluded. Studies were further excluded if they did not report sport performance outcomes, reported only physiological, biochemical, or perceptual outcomes, provided insufficient data for effect-size calculation, or had no eligible comparator. Reviews, conference abstracts, non-human studies, and duplicate reports based on the same dataset were excluded. When multiple reports were published from the same study, only the report with the most complete and relevant data was retained.

### 2.2 Data extraction

Two authors independently extracted and cross-checked data using a predesigned Microsoft Excel extraction form. Disagreements were resolved through discussion, and an independent third reviewer was consulted when necessary. Extracted information included author, year, country, sport, performance level, sample size, age, intervention and comparator, key intervention parameters, intervention-to-test interval, outcome category, outcome measure, and numerical data required for effect-size calculation. When numerical data were incomplete, the corresponding authors were contacted by email; when data were presented only graphically, WebPlotDigitizer was used to extract the required values.

All outcomes were categorized as explosive/jump, speed/change-of-direction, sport-specific, or endurance performance. Representative outcomes included jump and power tests, sprint and agility tests, sport-specific performance tests, and sustained-performance measures such as time to exhaustion, time-trial performance, and maximal accumulated oxygen deficit.

When multiple outcomes were reported within the same study, outcome selection rules were prespecified according to PRISMA-NMA and Cochrane Handbook recommendations for handling multiple outcomes and multi-arm studies (Hutton et al., 2015; Higgins et al., 2024). Outcomes were first assigned to the four predefined performance categories, and when multiple outcomes were available within the same category, the outcome most closely related to sport-specific performance was retained. For studies with multiple protocols of the same intervention type, only one representative protocol was included in the main analysis to avoid multiple contributions from the same study to the same outcome domain. Priority was given to the primary protocol specified in the original study; otherwise, the protocol that was more commonly used, more completely reported, and more comparable with other studies was selected.

When multiple post-intervention assessments were reported, one representative time point was selected for the main analysis, while the remaining assessments were recorded descriptively. Preferred time points were 7–10 min after PAPE (Wilson et al., 2013), approximately 45 min after IPC or RIPC (Salvador et al., 2016), immediately or shortly after stretching or FR (Behm and Chaouachi, 2011; Hendricks et al., 2020), and after passive heat maintenance following active warm-up (Cowper et al., 2022). When no assessment fell within the preferred window, the sole or nearest available time point was retained, as no fixed numerical tolerance had been prespecified. For regular warm-up and sham conditions, the time point matched to the corresponding intervention was used. These windows guided outcome selection rather than study eligibility and were intended to reduce selective extraction and improve comparability across studies.

Participant training and performance caliber was classified into six levels (Tier 0–5) according to the framework (McKay et al., 2022): sedentary, recreationally active, trained/developmental, highly trained/national-level, elite/international-level, and world-class athletes.

### 2.3 Risk of bias assessment

The risk of bias of the included studies was independently assessed by two authors using RevMan 5.4.1 and the Cochrane risk-of-bias tool (Higgins et al., 2011). This tool evaluates seven domains: random sequence generation, allocation concealment, blinding of participants and personnel, blinding of outcome assessment, incomplete outcome data, selective outcome reporting, and other potential sources of bias. Each domain was rated as having a low, high, or unclear risk of bias. Because most acute sport performance interventions, such as stretching, PAPE, foam rolling, and passive heat maintenance, have obvious operational characteristics, blinding of participants and intervention providers was generally difficult to implement and was therefore interpreted cautiously. Any disagreements between the two authors were resolved through discussion with an independent third reviewer.

### 2.4 Certainty of evidence assessment

The certainty of evidence for the main network estimates was assessed using Confidence in Network Meta-Analysis (CINeMA), which is based on the Grading of Recommendations Assessment, Development and Evaluation (GRADE) framework for network meta-analysis. The assessment was conducted for selected key comparisons within each outcome category. Because the included studies were randomized controlled trials or randomized crossover trials, the initial confidence in the evidence was considered high. CINeMA evaluates confidence across six domains: within-study bias, reporting bias, indirectness, imprecision, heterogeneity, and incoherence (Nikolakopoulou et al., 2020).

Within-study bias was informed by the Cochrane risk-of-bias assessment of the included studies. Indirectness was judged according to the consistency of participants, interventions, comparators, and outcomes with the predefined PICOS criteria. Reporting bias was assessed using comparison-adjusted funnel plots. Given that standardized mean differences (SMDs) were used as the effect measure, an SMD of 0.20 was predefined as the threshold for a practically important effect. Two authors independently conducted the CINeMA assessments, and disagreements were resolved through discussion with an independent third reviewer.

### 2.5 Statistical analysis

A frequentist random-effects network meta-analysis was performed using Stata 18.0. Considering the expected heterogeneity across included studies in sport type, key intervention parameters, post-intervention testing time points, and outcome measures, a random-effects model was used. Between-study heterogeneity was quantified using the common heterogeneity variance (τ²) estimated from the random-effects network meta-analysis. Approximate 95% confidence intervals for τ² were calculated where estimable. Because all outcomes were continuous variables and differed across studies in testing protocols and measurement units, SMDs with 95% confidence intervals (CIs) were used as effect-size measures. An SMD > 0 indicated improved sport performance. For outcomes in which lower values indicated better performance, the direction of the mean values was reversed before analysis to ensure consistency in effect direction across outcomes. For crossover trials, the main analysis used the reported sample sizes without applying an explicit within-participant correlation adjustment. For trials without reported within-participant correlations, sensitivity analyses were conducted using assumed correlation coefficients of 0.30, 0.50, and 0.70, consistent with previous meta-analytic practice (Hahn et al., 2024), to assess the robustness of the findings to alternative correlation assumptions.

Network plots were constructed according to outcome type. Nodes represented different interventions, node size reflected the sample size included for each intervention, edges represented direct comparisons between interventions, and edge thickness reflected the number of studies contributing to each direct comparison. A global inconsistency model was used to assess overall inconsistency. A P value > 0.05 was interpreted as indicating no significant inconsistency between direct and indirect evidence. Local inconsistency was assessed using the node-splitting method; a P value > 0.05 indicated no significant inconsistency between local direct and indirect evidence. When closed loops were present, loop-specific inconsistency was further assessed by calculating the inconsistency factor (IF) and its 95% CI to evaluate agreement between direct and indirect evidence within the loop. If the 95% CI of the IF included 0, no significant inconsistency within the closed loop was considered to be present.

Pairwise comparative effects between interventions were presented in league tables. For statistically significant network estimates, the number of direct studies (k) and unique participants (n) contributing to the corresponding direct comparison was reported. To further assess the relative advantages of different interventions, interventions were ranked using the surface under the cumulative ranking curve (SUCRA) and ranking probabilities. A higher SUCRA value indicated a greater probability that the intervention would rank favorably for the corresponding outcome. Comparison-adjusted funnel plots were generated to assess potential publication bias and small-study effects. Small-study effects were further assessed using Egger’s regression test based on comparison-adjusted effect estimates, with standard errors clustered by study.

## 3 Results

### 3.1 Study selection and general characteristics

The database search identified 3,410 records. After duplicates were removed using EndNote 21 and manual checking, 2,458 records remained for title and abstract screening. Of these, 432 records were retained for further assessment. Following additional eligibility screening and report retrieval, 207 records were excluded, leaving 225 full-text reports for eligibility assessment. During full-text assessment and data extraction, 136 reports were excluded because of ineligible participants, incomplete data, or inappropriate outcome measures. Finally, 89 publications contributing 90 independent datasets were included in the network meta-analysis. The flow of study identification, screening, eligibility assessment, and final inclusion is shown in **Figure 2**.

**Figure 2:**
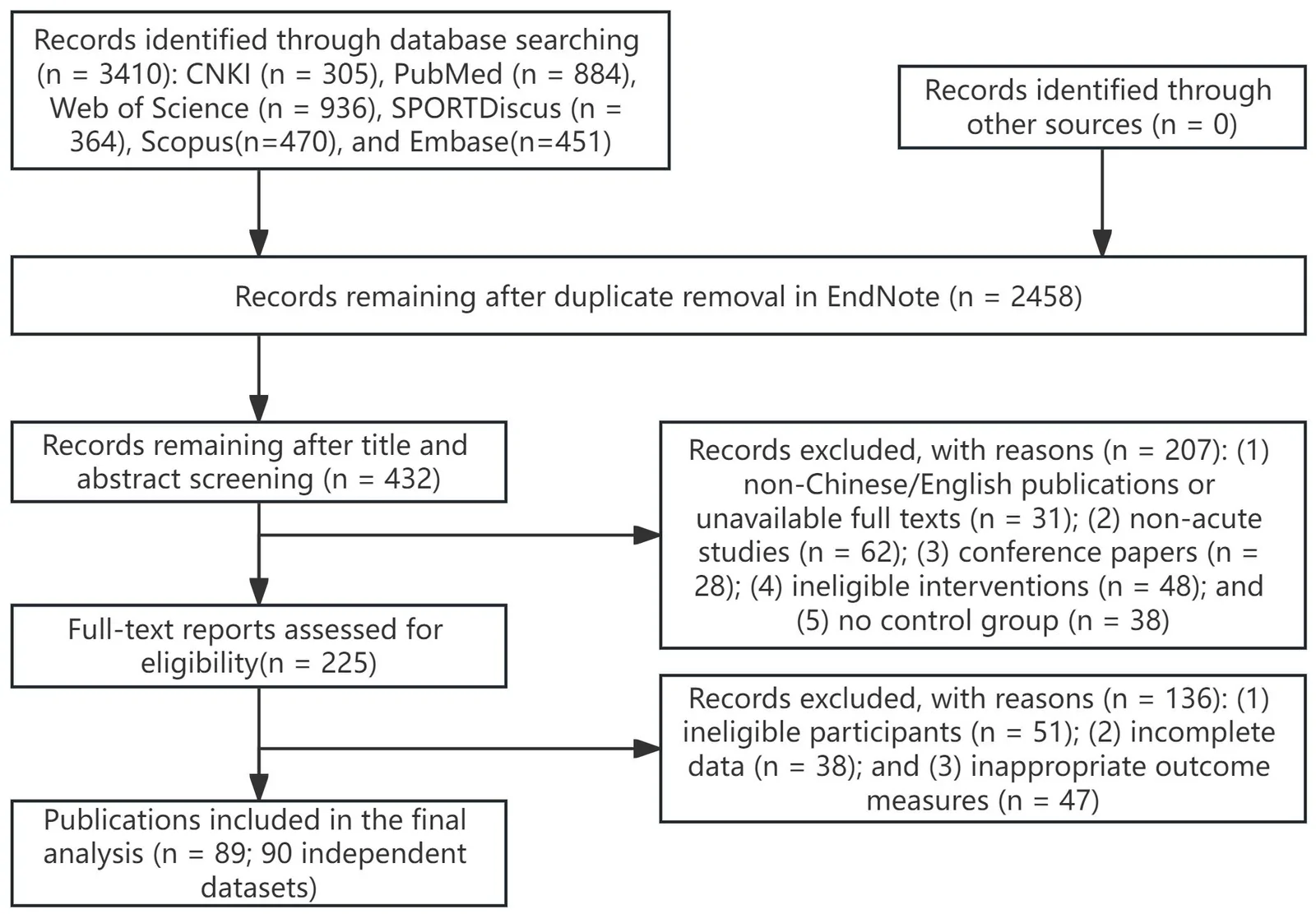
Flowchart of study selection.

The 89 included publications contributed 90 independent datasets involving 1,609 athletes. One included publication reported two independent randomized crossover trials involving separate participant samples and different performance-test protocols; therefore, these trials were treated as two independent datasets in the analyses. All included datasets were derived from randomized crossover trials or randomized controlled trials. The included sample consisted of 1,083 males and 402 females, while sex was not explicitly reported for 124 athletes. Athlete age ranged from 11 to 36 years. According to the participant classification framework (McKay et al., 2022), the included athletes were classified as Tier 2 (n = 974), Tier 3 (n = 265), or Tier 4 (n = 370), with no participants classified as Tier 0, Tier 1, or Tier 5. The mean training and performance caliber was 2.62 ± 0.83, indicating that the overall sample ranged from trained/developmental to highly trained/national-level athletes and could therefore be considered high-level athletes.

The included datasets covered four outcome categories: explosive/jump performance (n = 42), speed/change-of-direction performance (n = 36), sport-specific performance (n = 22), and endurance performance (n = 14). Because some datasets contributed outcomes to more than one performance category, these counts were not mutually exclusive. Explosive/jump performance mainly included countermovement jump (CMJ), vertical jump (VJ), squat jump (SJ), peak power, mean power, and medicine-ball throwing distance. Speed/change-of-direction performance mainly included short-distance sprint time or speed, repeated-sprint performance, 505 change-of-direction test (505 test), T-test agility, Illinois agility test, and zigzag test. Sport-specific performance mainly included kicking velocity, serve velocity, swimming performance, hitting distance, sport-specific throwing performance, and other sport-specific test outcomes. Endurance performance mainly included time to exhaustion, time-trial performance, maximal accumulated oxygen deficit, peak aerobic power, and sustained exercise distance.

The included studies involved 12 acute intervention and comparator conditions: regular warm-up (WU), post-activation performance enhancement (PAPE), ischemic preconditioning (IPC), remote ischemic preconditioning (RIPC), static stretching (SS), dynamic stretching (DS), foam rolling (FR), passive heat maintenance (HEAT), sham ischemic preconditioning (SHAM), static stretching followed by dynamic stretching (SS-DS), dynamic stretching followed by static stretching (DS-SS), and dynamic stretching combined with foam rolling (DS-FR). The characteristics of the included studies are presented in **Supplementary Table 1**.

### 3.2 Risk of bias assessment

The overall risk-of-bias distribution is presented in **Figure 3**, while study-level judgments for all 90 independent datasets are provided in **Supplementary Figure S1A–D**. Most datasets showed low risk of bias for random sequence generation and outcome assessment, but unclear allocation concealment and selective reporting were common. Blinding of participants and personnel was rated as high risk in most studies because acute interventions such as PAPE, stretching, foam rolling, and passive heat maintenance were difficult to blind. For other sources of bias, most studies were rated as low risk.

**Figure 3:**
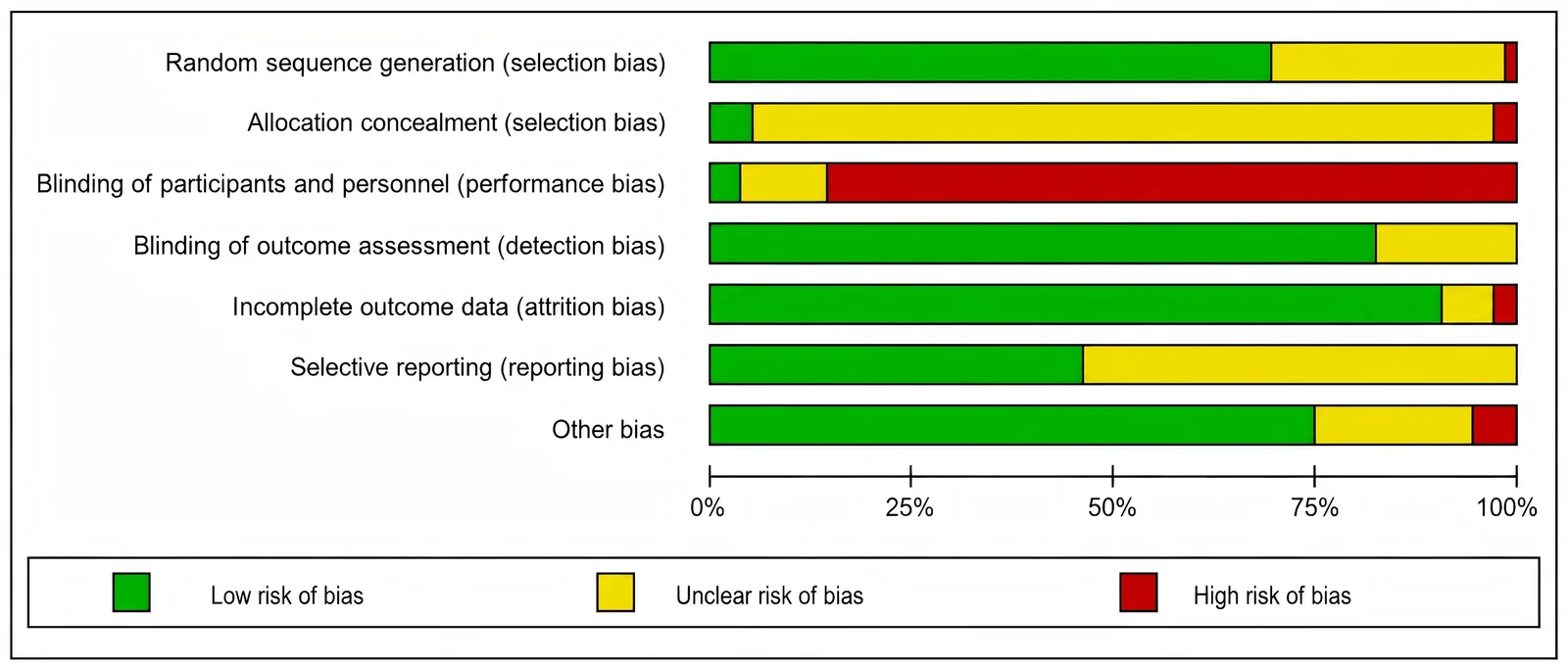
Overall risk-of-bias summary for the included datasets. Red indicates a high risk of bias, green indicates a low risk of bias, and yellow indicates an unclear risk of bias.

### 3.3 Network meta-analysis results

#### 3.3.1 Network heterogeneity and consistency

τ² was 0.014 (approximate 95% CI: 0.001–0.394) for explosive/jump performance and τ² < 0.001 for speed/change-of-direction, sport-specific, and endurance performance; 95% CIs for the latter three networks were not estimable because the τ² estimates were at the boundary of zero. Detailed between-study heterogeneity estimates are presented in **Supplementary Table S2**. No significant global inconsistency was detected in any of the four networks: explosive/jump performance (P = 0.9137), speed/change-of-direction performance (P = 0.8988), sport-specific performance (P = 0.2252), and endurance performance (P = 0.6187). Node-splitting analyses showed no significant local inconsistency, with all P values > 0.05. Loop-specific inconsistency analyses also indicated no significant inconsistency. For explosive/jump performance, IF values ranged from 0.000 to 0.626; for speed/change-of-direction performance, IF values ranged from 0.006 to 0.379; for sport-specific performance, the IF values of the two closed loops were 0.044 and 1.044; and for endurance performance, IF values ranged from 0.112 to 0.917. In all closed loops, P values were > 0.05 and the 95% CIs of the IF included 0. Therefore, the consistency model was used for the main analyses.

#### 3.3.2 Explosive/jump performance

A total of 42 datasets were included for explosive/jump performance, involving 11 intervention/comparator conditions: PAPE, HEAT, DS, FR, IPC, SS-DS, DS-SS, WU, DS-FR, SHAM, and SS. The network evidence plot is shown in **Figure 4A**.

**Figure 4:**
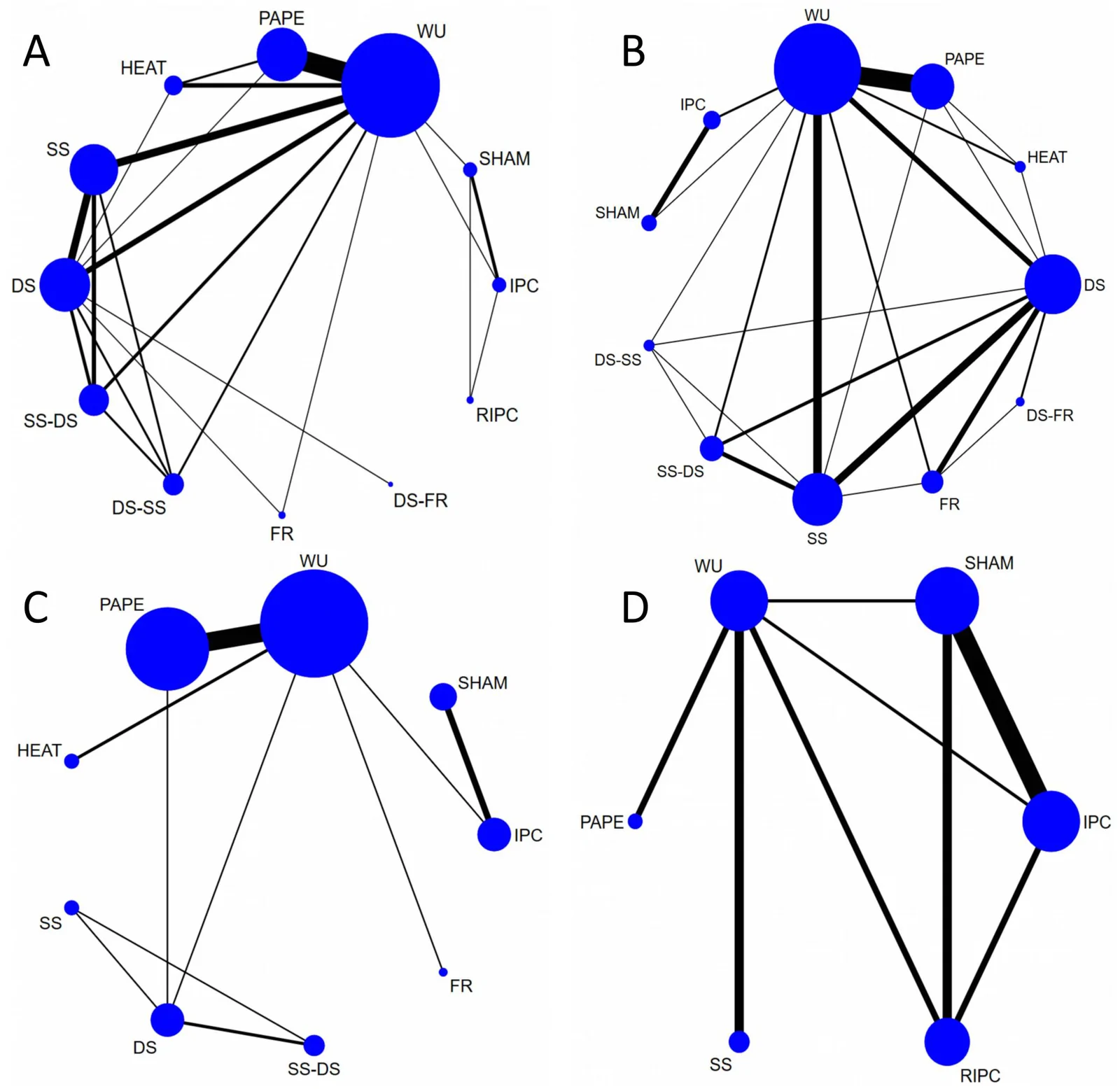
Network evidence plots for acute pre-competition conditioning strategies. **(A)** explosive/jump performance; **(B)** speed/change-of-direction performance; **(C)** sport-specific performance; **(D)** endurance performance. Nodes represent interventions, node size reflects sample size, edges represent direct comparisons, and edge thickness reflects the number of studies. WU, regular warm-up; PAPE, post-activation performance enhancement; SS, static stretching; DS, dynamic stretching; FR, foam rolling; HEAT, passive heat maintenance; IPC, ischemic preconditioning; RIPC, remote ischemic preconditioning; SHAM, sham ischemic preconditioning. SS-DS, static stretching followed by dynamic stretching; DS-SS, dynamic stretching followed by static stretching; DS-FR, dynamic stretching combined with foam rolling.

PAPE was significantly superior to WU [SMD = 0.40, 95% CI 0.20–0.59; k = 16, n = 239] and SS [SMD = 0.60, 95% CI 0.32–0.88; k = 1, n = 14]. DS [SMD = 0.38, 95% CI 0.15–0.60; k = 7, n = 138] and FR [SMD = 0.36, 95% CI 0.05–0.68; k = 1, n = 23] were also significantly superior to SS. The remaining pairwise comparisons were not statistically significant because their 95% CIs crossed zero (**Figure 5A**). The SUCRA-based ranking of intervention effects was as follows: PAPE (91.5%) > HEAT (74.2%) > DS (66.3%) > FR (62.9%) > IPC (58.4%) > SS-DS (46.3%) > WU (37.6%) > DS-SS (36.5%) > SHAM (33.9%) > DS-FR (30.4%) > SS (11.9%) (**Figure 6A**). Although HEAT ranked second (SUCRA = 74.2%), this ranking was supported by only two direct studies involving 35 athletes and should therefore be interpreted cautiously.

**Figure 5:**
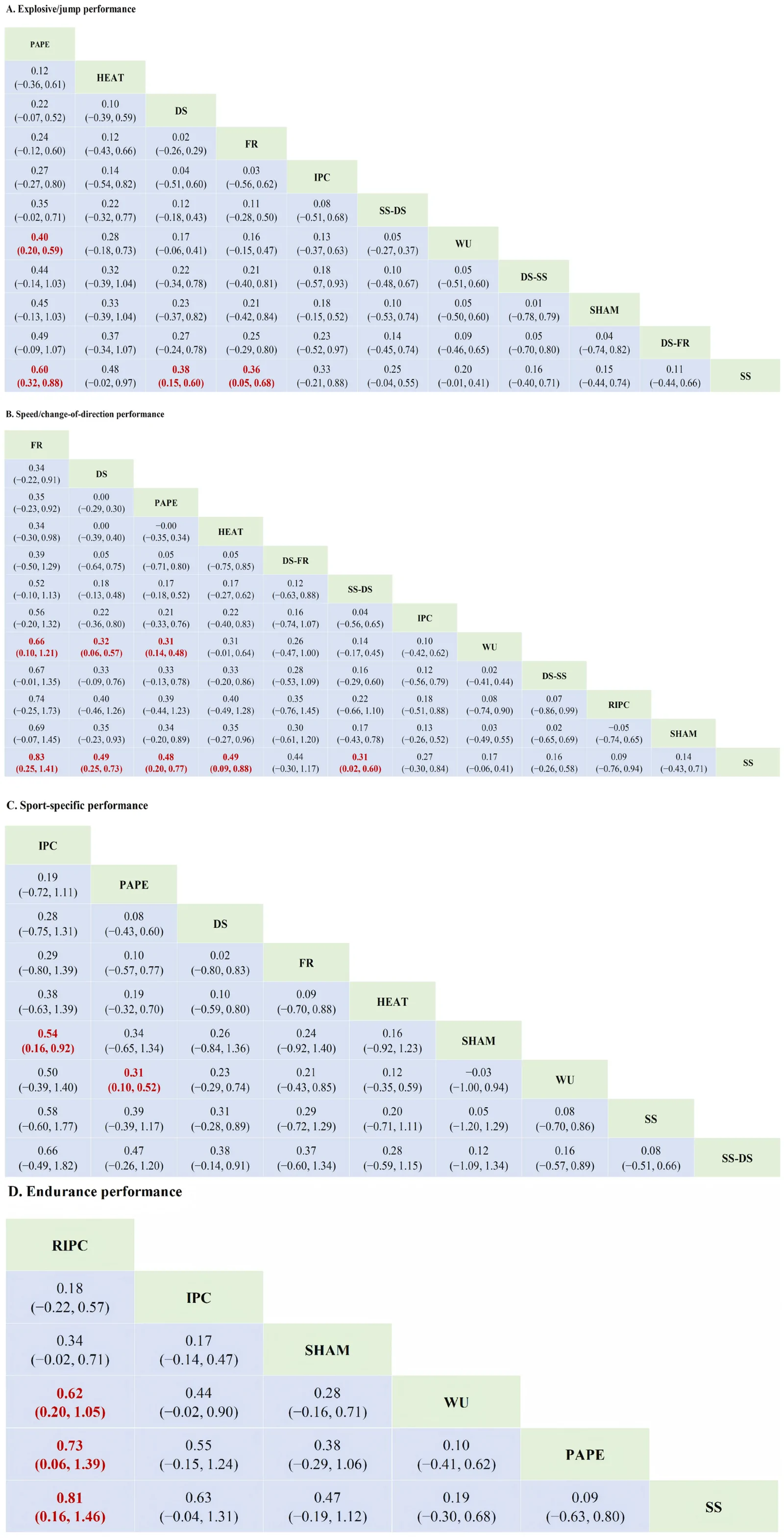
League tables for the effects of acute pre-competition conditioning strategies. **(A)** explosive/jump performance; **(B)** speed/change-of-direction performance; **(C)** sport-specific performance; **(D)** endurance performance. Values are standardized mean differences (SMDs) with 95% confidence intervals (CIs) for column versus row treatments. Positive values favor the column treatment, and significant effects are shown in bold red.

**Figure 6:**
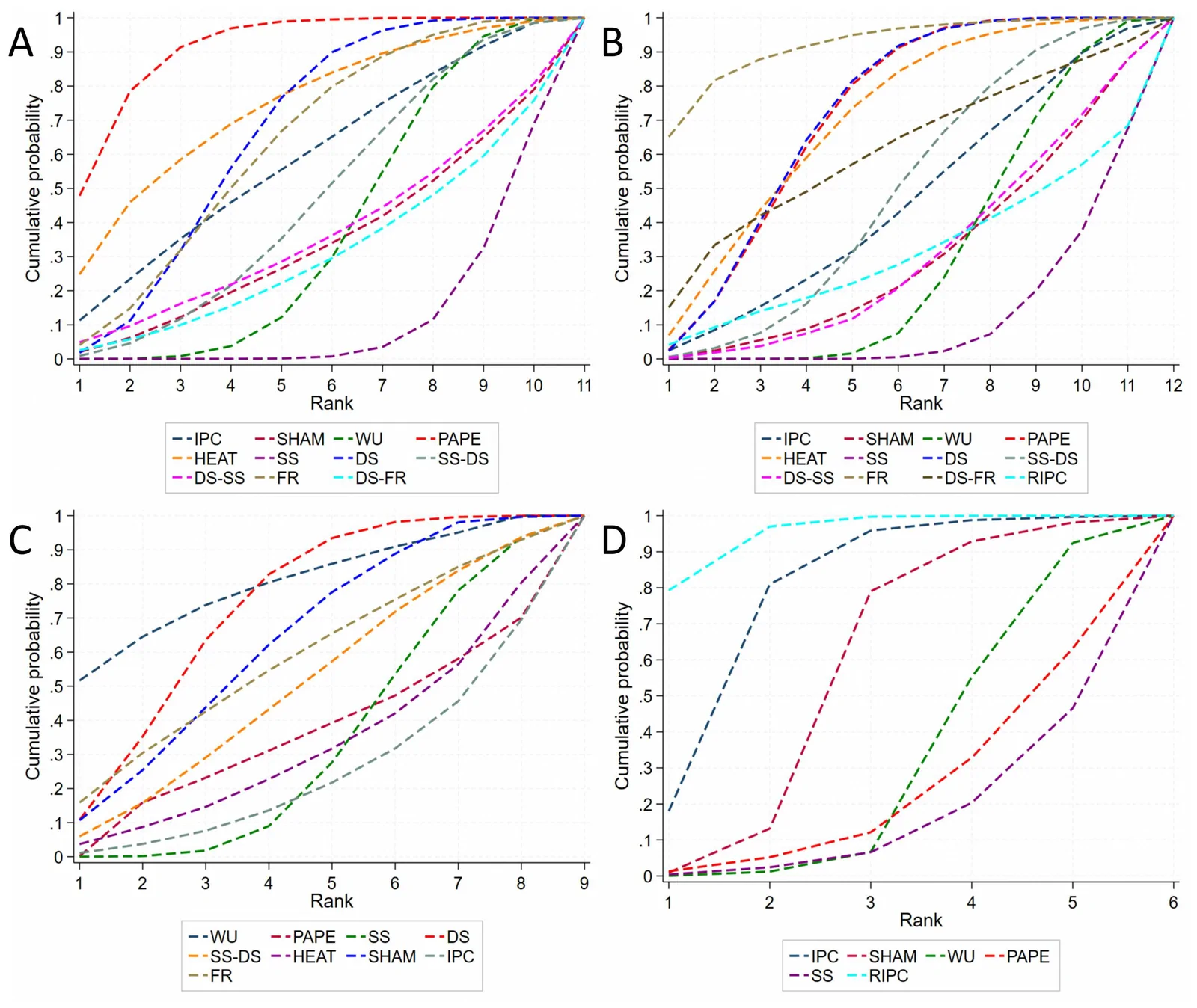
Cumulative ranking probability curves for acute pre-competition conditioning strategies. **(A)** explosive/jump performance; **(B)** speed/change-of-direction performance; **(C)** sport-specific performance; **(D)** endurance performance. A larger area under the curve indicates a greater probability of a favorable ranking.

#### 3.3.3 Speed/change-of-direction performance

A total of 36 datasets were included for speed/change-of-direction performance, involving 12 intervention/comparator conditions: PAPE, HEAT, DS, FR, IPC, SS-DS, DS-SS, WU, DS-FR, SHAM, SS, and RIPC. The network evidence plot is shown in **Figure 4B**.

FR [SMD = 0.66, 95% CI 0.10–1.21; k = 1, n = 16], PAPE [SMD = 0.31, 95% CI 0.14–0.48; k = 18, n = 290], and DS [SMD = 0.32, 95% CI 0.06–0.57; k = 5, n = 76] were significantly superior to WU. Compared with SS, FR [SMD = 0.83, 95% CI 0.25–1.41; k = 0], PAPE [SMD = 0.48, 95% CI 0.20–0.77; k = 0], and HEAT [SMD = 0.49, 95% CI 0.09–0.88; k = 0] showed significant favorable effects based entirely on indirect evidence. DS [SMD = 0.49, 95% CI 0.25–0.73; k = 7, n = 115] and SS-DS [SMD = 0.31, 95% CI 0.02–0.60; k = 4, n = 78] were also significantly superior to SS. The remaining pairwise comparisons were not statistically significant because their 95% CIs crossed zero (**Figure 5B**). The SUCRA-based ranking of intervention effects was as follows: FR (92.4%) > DS (72.3%) > PAPE (71.8%) > HEAT (70.4%) > DS-FR (61.1%) > SS-DS (49.6%) > IPC (46.3%) > WU (31.1%) > DS-SS (30.8%) > RIPC (30.6%) > SHAM (30.5%) > SS (12.4%) (**Figure 6B**). Although FR ranked first (SUCRA = 92.4%), the FR-versus-WU estimate was supported by only one direct study involving 16 athletes and was rated as moderate confidence.

#### 3.3.4 Sport-specific performance

A total of 22 datasets were included for sport-specific performance, involving nine intervention/comparator conditions: PAPE, HEAT, DS, FR, IPC, SS-DS, WU, SHAM, and SS. The network evidence plot is shown in **Figure 4C**.

IPC was significantly superior to SHAM [SMD = 0.54, 95% CI 0.16–0.92; k = 4, n = 56], and PAPE was significantly superior to WU [SMD = 0.31, 95% CI 0.10–0.52; k = 12, n = 181]. The remaining pairwise comparisons were not statistically significant because their 95% CIs crossed zero (**Figure 5C**). The SUCRA-based ranking of intervention effects was as follows: IPC (80.3%) > PAPE (73.2%) > DS (64.0%) > FR (57.5%) > HEAT (48.7%) > SHAM (36.2%) > WU (33.6%) > SS (31.9%) > SS-DS (24.6%) (**Figure 6C**). Although DS ranked third (SUCRA = 64.0%), the DS-versus-WU comparison was supported by only one direct study involving 23 athletes and was rated as very low confidence.

#### 3.3.5 Endurance performance

A total of 14 datasets were included for endurance performance, involving six intervention/comparator conditions: PAPE, IPC, WU, RIPC, SHAM, and SS. The network evidence plot is shown in **Figure 4D**.

RIPC was significantly superior to WU [SMD = 0.62, 95% CI 0.20–1.05; k = 2, n = 40], PAPE [SMD = 0.73, 95% CI 0.06–1.39; k = 0], and SS [SMD = 0.81, 95% CI 0.16–1.46; k = 0]. The remaining pairwise comparisons were not statistically significant because their 95% CIs crossed zero (**Figure 5D**). The SUCRA-based ranking of intervention effects was as follows: RIPC (95.1%) > IPC (78.3%) > SHAM (57.1%) > WU (31.2%) > PAPE (20.9%) > SS (15.5%) (**Figure 6D**). The favorable rankings of RIPC and IPC were based on only two direct studies involving 40 athletes and one direct study involving 24 athletes versus WU, respectively. Moreover, the RIPC-versus-PAPE and RIPC-versus-SS estimates were based entirely on indirect evidence; therefore, these rankings should be interpreted cautiously.

### 3.4 Publication bias and small-study effects

Comparison-adjusted funnel plots and Egger’s regression tests were used to assess potential publication bias and small-study effects (**Figure 7**). Egger’s tests showed no significant small-study effects for explosive/jump performance (P = 0.704), speed/change-of-direction performance (P = 0.100), sport-specific performance (P = 0.561), or endurance performance (P = 0.197).

**Figure 7:**
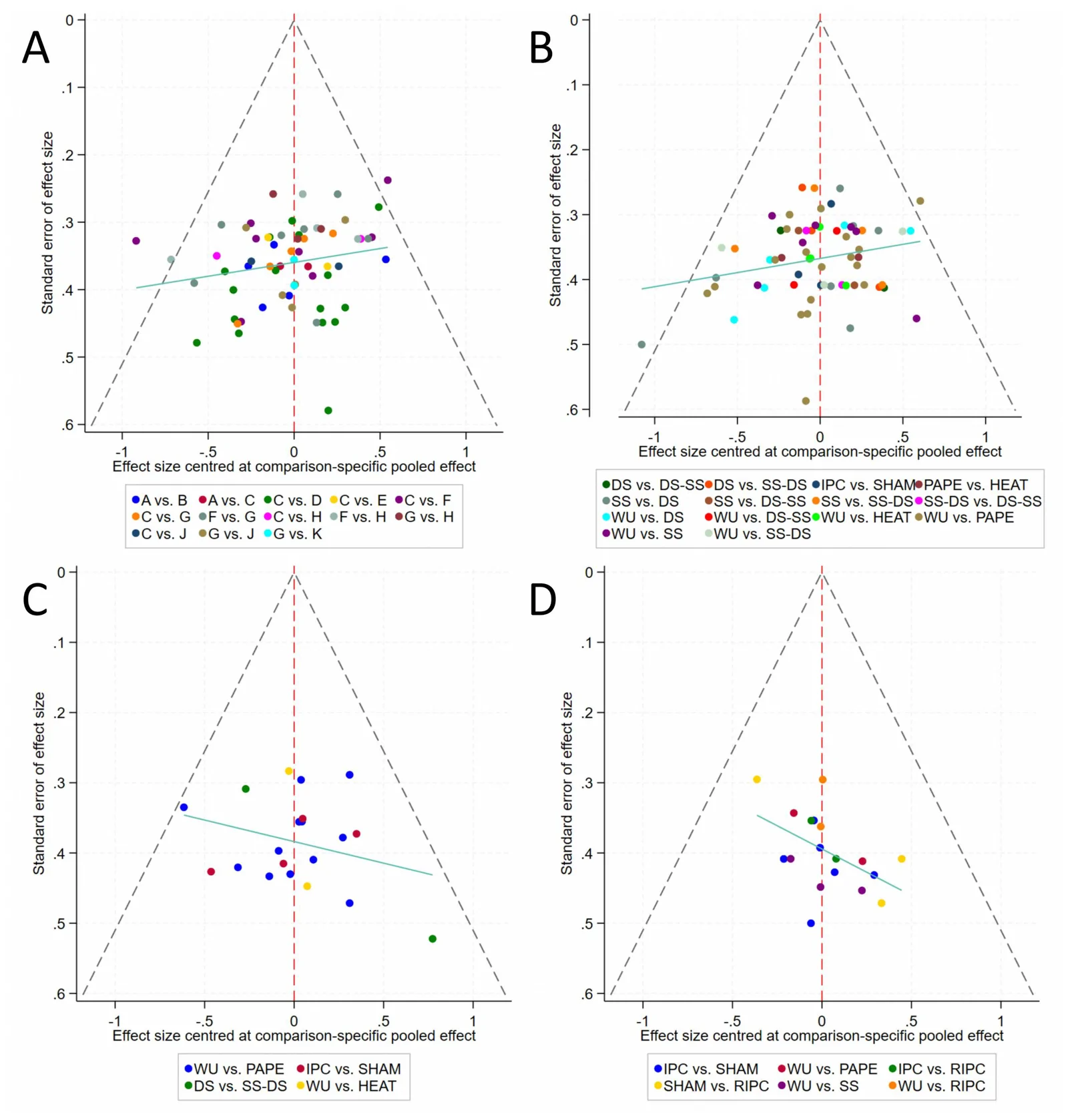
Comparison-adjusted funnel plots for the effects of acute pre-competition conditioning strategies on sport performance. **(A)** explosive/jump performance; **(B)** speed/change-of-direction performance; **(C)** sport-specific performance; **(D)** endurance performance. Treatment codes: A = IPC, B = SHAM, C = PAPE, D = WU, E = DS, F = DS-SS, G = SS, H = SS-DS, I = HEAT, J = DS-FR, K = FR, and L = RIPC. Funnel-plot asymmetry was further assessed using Egger’s regression tests based on comparison-adjusted effect estimates; detailed results are presented in **Supplementary Table S3**.

### 3.5 Sensitivity analyses

Sensitivity analyses using assumed within-participant correlations of 0.30, 0.50, and 0.70 showed no material changes in the direction, statistical significance, or interpretation of the main comparisons. The SUCRA-based top-three rankings were also stable, except for a minor change in the speed/change-of-direction network at r = 0.30, where HEAT replaced PAPE in the top three. Full results are provided in **Supplementary Tables S8**-**S10**.

### 3.6 Certainty of evidence assessment

CINeMA confidence ratings for selected key comparisons from each outcome network are summarized in **Table 1**, while complete assessments across all six CINeMA domains are provided in the **Supplementary Material**.

**Table 1:** Summary of CINeMA confidence ratings for selected key network comparisons.

Outcome network	Comparison	SMD (95% CI)	Direct studies, k	CINeMA confidence
Explosive/jump	PAPE vs WU	0.40 (0.20–0.59)	16	High
Speed/COD	PAPE vs WU	0.31 (0.14–0.48)	18	High
Sport-specific	IPC vs SHAM	0.54 (0.16–0.92)	4	High
Endurance	RIPC vs WU	0.62 (0.20–1.05)	2	High

CI, confidence interval; CINeMA, Confidence in Network Meta-Analysis; COD, change of direction; IPC, ischemic preconditioning; PAPE, post-activation performance enhancement; RIPC, remote ischemic preconditioning; SHAM, sham stimulation; SMD, standardized mean difference; WU, regular warm-up; k, number of direct studies. Positive SMDs favor the first-listed intervention.

Across the four outcome networks, CINeMA confidence ratings varied considerably. High-confidence evidence supported PAPE versus WU for explosive/jump and speed/change-of-direction performance, IPC versus SHAM for sport-specific performance, and RIPC versus WU for endurance performance. Other comparisons within the endurance network were generally less certain. Across the complete assessments, downgrading was mainly driven by imprecision and indirectness, while heterogeneity affected selected comparisons. Within-study bias and reporting bias generally raised few concerns.

## 4 Discussion

### 4.1 Outcome specificity and strategy matching

This network meta-analysis indicates that acute pre-competition conditioning strategies produce outcome-specific rather than universal performance benefits. PAPE appeared most applicable to explosive/jump performance; FR, DS, and PAPE were relatively favorable for speed/change-of-direction performance; IPC and PAPE showed advantages for sport-specific performance; and RIPC and IPC ranked higher for endurance performance. Strategy selection should therefore be matched to the target performance demand, athlete characteristics, and competition context rather than based solely on the overall intervention ranking.

The practical magnitude of these findings should be interpreted within the context of high-level sport. Statistically significant estimates against WU or SHAM ranged from 0.31 to 0.66, representing generally small-to-moderate standardized effects. Although modest in conventional statistical terms, such effects may still be competitively meaningful because the margins separating high-level athletes are often small. Hopkins et al. (1999) suggested that the smallest worthwhile performance enhancement in international athletes may correspond to approximately 0.4–0.7 times the typical within-athlete variation between competitions, while Pyne et al. (2004) reported that an additional improvement of approximately 0.4% could meaningfully increase medal prospects in Olympic swimmers. However, because the included studies used heterogeneous tests and measurement scales, the observed SMDs cannot be directly translated into seconds, centimeters, points, or competition rankings. Their practical relevance is most likely when the improvement exceeds normal performance variability and is reproducible without introducing additional fatigue.

For explosive/jump performance, PAPE was superior to WU and SS and showed the most favorable overall findings. An appropriately prescribed conditioning activity may improve subsequent force production by increasing neuromuscular activation, recruitment of high-threshold motor units, and muscle temperature (Wilson et al., 2013). Liu et al. (2024) reported small favorable effects of resistance-based conditioning activities on jump performance (Hedges’ g = 0.20–0.23), with resistance-based protocols generally showing larger effects than plyometric or combined protocols. In the present NMA, PAPE versus WU produced an SMD of 0.40, although direct numerical comparison is limited by differences in effect measures, comparators, and analytical methods. However, the balance between performance enhancement and residual fatigue may be influenced by conditioning load, exercise volume, movement specificity, recovery interval, and individual strength capacity. PAPE protocols should therefore be individualized rather than applied uniformly across athletes and sports.

For speed/change-of-direction performance, PAPE and DS were favorable compared with WU. DS may increase muscle temperature and range of motion while maintaining neuromuscular activation, making it more suitable than prolonged SS immediately before sprinting or change-of-direction tasks. Li et al. (2023) reported that DS increased countermovement-jump height by 1.60 cm (95% CI 0.67–2.60) and reduced sprint time by 0.08 s (95% CI −0.15 to −0.008). Consistent with these findings, the present NMA favored DS over WU for speed/change-of-direction performance (SMD = 0.32) and over SS for explosive/jump performance (SMD = 0.38). Nevertheless, the numerical estimates should not be directly compared because the reviews used different effect measures, comparator conditions, populations, and outcome classifications.

FR also received a favorable ranking for speed/change-of-direction performance. Its potential benefits may include increased range of motion, reduced perceived stiffness, and improved subjective readiness. However, evidence that these changes consistently improve force, power, or sprint performance remains inconsistent (Hendricks et al., 2020). Therefore, FR should not be considered superior to PAPE or DS solely on the basis of its SUCRA ranking and may be more appropriately used as a brief adjunct to dynamic or sport-specific activation.

IPC and RIPC ranked relatively highly for sport-specific and endurance performance. Repeated ischemia-reperfusion cycles may influence blood flow, oxygen utilization, and metabolite handling (Salvador et al., 2016), which may be more relevant to sustained or mixed aerobic–anaerobic activities than to isolated explosive tasks. Zhang et al. (2026) reported a small overall effect size of IPC on sport performance (0.13, 95% CI 0.06–0.21) and found no significant benefit among highly trained, elite, or world-class athletes. Similarly, Souza et al. (2025) found that IPC was generally comparable to a low-pressure placebo across performance outcomes, except for a small improvement in time to exhaustion (SMD = 0.37, P = 0.002). These findings support cautious interpretation of the present IPC and RIPC rankings, as responses may depend on training level, placebo or expectancy effects, cuff protocol, and intervention timing. These strategies should therefore be tested individually before competition.

Passive heat maintenance may help preserve the temperature-related benefits of active warm-up when a prolonged delay occurs before performance (McGowan et al., 2015; Cowper et al., 2022). Heated garments may be useful during check-in procedures, substitution periods, or intervals between competitive rounds. However, their effectiveness depends on waiting duration, environmental temperature, and the risk of excessive thermal strain (Menzies et al., 2025). HEAT should therefore be considered a context-specific strategy rather than a routine intervention for all athletes.

Importantly, SUCRA reflects the probability of a favorable relative ranking rather than the magnitude, precision, certainty, or practical importance of an effect. Rankings should be interpreted together with confidence intervals, directness of evidence, and CINeMA confidence ratings (Nikolakopoulou et al., 2020), particularly when networks are sparse or evidence confidence is limited. Overall, the findings support an outcome-specific and individualized approach rather than a single universally optimal conditioning strategy.

### 4.2 Practical implications

The practical significance of this study lies not in providing a fixed hierarchy of acute pre-competition conditioning strategies, but in supporting more precise strategy selection according to performance type, competition context, and athlete profile. Therefore, the rankings should be interpreted as the relative application potential of different strategies, rather than as standardized warm-up templates that can be mechanically applied across sports. In practice, the goal should be to establish a minimum effective preparation routine that matches sport-specific demands without introducing additional fatigue or discomfort. **Table 2** provides a concise practice-oriented summary of the recommended strategies, target outcomes, suggested intervals, population evidence, practical applications, and representative CINeMA confidence ratings.

**Table 2:** Strategy selection guide for different acute pre-competition conditioning strategies.

Target outcome	Recommended strategy and confidence	Suggested interval	Population evidence	Practical considerations
Explosive/jump performance	PAPE (High vs WU); DS as an adjunct (Moderate vs WU)	PAPE: 7–10 min; DS: immediately or shortly before performance	Evidence for PAPE and DS included both adolescent and adult athletes and covered male, female, and mixed-sex samples, suggesting relatively broad population representation.	Most relevant to jump-, power-, and explosive-force-dominant tasks when sufficient recovery time is available.
Speed/change-of-direction performance	PAPE (High vs WU) or DS (High vs WU); FR as an adjunct (Moderate vs WU)	PAPE: 7–10 min; DS/FR: immediately or shortly before performance	PAPE and DS were represented across adolescent and adult athletes and included both male and female samples. In contrast, FR evidence was sparse and derived only from male samples.	Applicable to sprint, acceleration, agility, and change-of-direction tasks; FR should be used cautiously because of limited direct evidence.
Sport-specific performance	PAPE (High vs WU) or IPC (High vs SHAM; Very low vs WU)	PAPE: 7–10 min; IPC: approximately 45 min	PAPE had broader age and sex representation, whereas the available IPC evidence for sport-specific performance was limited to adult male athletes.	PAPE may be considered when the conditioning activity closely matches the target sport task; IPC requires a substantially longer pre-performance window.
Endurance performance	RIPC (High vs WU) or IPC (Low vs WU)	Approximately 45 min	IPC/RIPC evidence was derived from adult athletes, with limited female representation and no predominantly adolescent datasets.	Potentially applicable to sustained-performance events when an approximately 45-min pre-start window is feasible.

CINeMA, Confidence in Network Meta-Analysis; DS, dynamic stretching; FR, foam rolling; IPC, ischemic preconditioning; PAPE, post-activation performance enhancement; RIPC, remote ischemic preconditioning; SHAM, sham stimulation; WU, regular warm-up. CINeMA confidence ratings are comparison- and outcome-specific and should not be interpreted as overall confidence ratings for individual interventions.The suggested intervals should be regarded as practical starting points rather than fixed prescriptions because intervention protocols and assessment timings varied across studies. Recommendations should be interpreted alongside their CINeMA confidence ratings, and each strategy should be tested and individualized under training or simulated-competition conditions before use in formal competition.

### 4.3 Limitations and future directions

This study has several limitations. First, the included studies differed in sport discipline, athlete caliber, developmental stage, intervention protocol, recovery interval, and outcome assessment. Although the observed τ² estimates were generally low, this does not eliminate clinical and methodological heterogeneity. Participants ranged from 11 to 36 years, and combining adolescent and adult athletes may have introduced differences related to growth and maturation. Age, sex, sport type, competition level, and intervention timing could not be examined as effect modifiers because reporting was incomplete and most comparisons contained too few studies for reliable subgroup analyses or meta-regression.

Second, the participants were predominantly male athletes classified within McKay Tiers 2–4, with no Tier 5 world-class athletes represented. Therefore, the findings should be generalized cautiously to female athletes, world-class athletes, recreational populations, and specific developmental stages. The sport-specific and endurance networks were also relatively sparse, with some estimates relying substantially on indirect evidence. Egger’s tests did not indicate significant small-study effects in any of the four networks, although nonsignificant tests do not completely exclude publication bias.

Third, within-participant correlations were not consistently reported in crossover trials, requiring assumed coefficients in sensitivity analyses. Blinding was difficult for several physical interventions, and expectancy or implementation effects may have influenced IPC and RIPC. The review was restricted to English- and Chinese-language publications, which may have introduced language bias. Finally, most studies were conducted under controlled testing conditions rather than formal competition. Future studies should use adequately powered direct comparisons, improve reporting of athlete and intervention characteristics, and include more female, youth, and world-class athletes under realistic competition conditions.

## 5 Conclusions

This network meta-analysis showed that the effects of acute pre-competition conditioning strategies in high-level athletes were outcome-specific rather than universally consistent across performance domains. PAPE showed the greatest application potential for explosive/jump performance, whereas FR, DS, and PAPE were more favorable for speed/change-of-direction performance. IPC and PAPE showed relative advantages for sport-specific performance, while RIPC and IPC ranked higher for endurance performance.

Overall, these findings indicate that the selection of acute pre-competition conditioning strategies should be guided by the target performance domain, competition context, intervention parameters, and individual athlete responses. Acute pre-competition conditioning should therefore be considered a sport-specific and individualized preparation tool rather than a standardized warm-up template.

Future research should use high-quality randomized crossover trials, rigorous comparator and sham-controlled conditions, and real competition settings to clarify which athletes, sports, timing windows, and intervention doses are most likely to benefit from these strategies.
