# Supplementary material for: Effects of pre-competition conditioning strategies on acute sport performance in high-level athletes: a systematic review and network meta-analysis
Source: Front Physiol. 2026 Oct 1;17:1930250. doi: 10.3389/fphys.2026.1930250 (PMC13669563; doi:10.3389/fphys.2026.1930250)
Supplement: Supplementary file 1 [file Supplementaryfile1.docx]

Supplementary Material

[Supplementary Section S1. Search Strategies and Database Yields	2](#_Toc14861)

[S1.1 Search period	2](#_Toc22784)

[S1.2 Database search yields	2](#_Toc3725)

[S1.3 Full database-specific search strategies	2](#_Toc24809)

[S1.3.1 PubMed	2](#_Toc21054)

[S1.3.2 Web of Science	3](#_Toc16559)

[S1.3.3 SPORTDiscus	4](#_Toc31488)

[S1.3.4 CNKI	4](#_Toc5199)

[S1.3.5 Scopus	6](#_Toc9227)

[S1.3.6 Embase	6](#_Toc1165)

[Supplementary Section S2. Risk-of-Bias Judgments	8](#_Toc3030)

[Supplementary Section S3. Additional Network Meta-Analysis Diagnostics	12](#_Toc13243)

[S3.1 Between-study heterogeneity	12](#_Toc6719)

[S3.2 Small-study effects	12](#_Toc17866)

[Supplementary Section S4. CINeMA Confidence Assessments	13](#_Toc30931)

[S4.1 Explosive/jump performance	13](#_Toc1519)

[S4.2 Speed/change-of-direction performance	14](#_Toc22114)

[S4.3 Sport-specific performance	15](#_Toc32353)

[S4.4 Endurance performance	16](#_Toc30800)

[Supplementary Section S5. Sensitivity Analyses	17](#_Toc15088)

[S5.1 Assumed within-participant correlations	17](#_Toc3896)

[S5.2 Sensitivity analyses of key network estimates	17](#_Toc30098)

[S5.3 Stability of intervention rankings	18](#_Toc12767)

[Supplementary Section S6. Abbreviations	19](#_Toc9238)

# Supplementary Section S1. Search Strategies and Database Yields

## S1.1 Search period

The six databases were searched from inception to 4 August 2026 using the predefined database-specific search strategies and eligibility criteria.

## S1.2 Database search yields

**Supplementary Table S1. Database search yields**

| **Database** | **Records identified** |
| --- | --- |
| PubMed | 884 |
| Web of Science | 936 |
| SPORTDiscus | 364 |
| CNKI | 305 |
| Scopus | 470 |
| Embase | 451 |
| Total | 3410 |

## S1.3 Full database-specific search strategies

## S1.3.1 PubMed

### Strategy 1. PAPE/PAP

**Records retrieved: 127**

( "Athletes"[Mesh] OR Athletes OR Athlete OR "Professional Athletes" OR "Athlete, Professional"OR "Athletes, Professional" OR "Professional Athlete" OR "Elite Athletes" OR "Athlete, Elite"OR "Athletes, Elite" OR "Elite Athlete" OR "well-trained" OR "well trained")
AND
("Athletic Performance"[Mesh] OR "Athletic Performance" OR "Athletic Performances" OR "Performance, Athletic"OR "Performance, Sports" OR "Performances, Athletic" OR "Performances, Sports"OR "Sports Performance" OR "Sports Performances"OR sport* OR athletic* OR exercis*)
AND
("post-activation potentiation"[Title/Abstract]OR PAP[Title/Abstract]OR "post activation performance enhancement"[Title/Abstract]OR PAPE[Title/Abstract]OR "conditioning contraction"[Title/Abstract]
OR "conditioning activity"[Title/Abstract]OR "conditioning activities"[Title/Abstract]OR "complex training"[Title/Abstract] )

### Strategy 2. Stretching and foam rolling

**Records retrieved: 596**

( "Athletes"[Mesh] OR Athletes OR Athlete OR "Professional Athletes" OR "Athlete, Professional"OR "Athletes, Professional" OR "Professional Athlete" OR "Elite Athletes" OR "Athlete, Elite"OR "Athletes, Elite" OR "Elite Athlete" OR "well-trained" OR "well trained")
AND
("Athletic Performance"[Mesh] OR "Athletic Performance" OR "Athletic Performances" OR "Performance, Athletic"OR "Performance, Sports" OR "Performances, Athletic" OR "Performances, Sports"OR "Sports Performance" OR "Sports Performances"OR sport* OR athletic* OR exercis*)
AND
("Static-Stretching" OR "Dynamic-Stretching" OR "Ballistic-Stretching"OR "Proprioceptive Neuromuscular Facilitation" OR "PNF-Stretching"OR "Static-Dynamic Stretching" OR "Combine of Stretching"OR "Foam Rolling" OR "Different stretching methods")

### Strategy 3. Passive heat maintenance

**Records retrieved: 25**

( "Athletes"[Mesh] OR Athletes OR Athlete OR "Professional Athletes" OR "Athlete, Professional"OR "Athletes, Professional" OR "Professional Athlete" OR "Elite Athletes" OR "Athlete, Elite"OR "Athletes, Elite" OR "Elite Athlete" OR "well-trained" OR "well trained")
AND
("Athletic Performance"[Mesh] OR "Athletic Performance" OR "Athletic Performances" OR "Performance, Athletic"OR "Performance, Sports" OR "Performances, Athletic" OR "Performances, Sports"OR "Sports Performance" OR "Sports Performances"OR sport* OR athletic* OR exercis*)
AND
("Passive Heat Maintenance" OR "passive heating" OR "heat maintenance" OR "insulated clothing" OR "warm up maintenance" OR "warm-up maintenance"OR "warmup maintenance" OR "thermal clothing" OR "warming garment*" )

### Strategy 4. IPC/RIPC

**Records retrieved: 136**

( "Athletes"[Mesh] OR Athletes OR Athlete OR "Professional Athletes" OR "Athlete, Professional"OR "Athletes, Professional" OR "Professional Athlete" OR "Elite Athletes" OR "Athlete, Elite"OR "Athletes, Elite" OR "Elite Athlete" OR "well-trained" OR "well trained")
AND
( "Athletic Performance"[Mesh] OR "Athletic Performance" OR "Athletic Performances" OR "Performance, Athletic"OR "Performance, Sports" OR "Performances, Athletic" OR "Performances, Sports"OR "Sports Performance" OR "Sports Performances"OR sport* OR athletic* OR exercis*)
AND
( "Ischemic Preconditioning"[Mesh] OR (Ischemic Preconditioning) OR (Ischemic Pre Conditioning) OR (Ischemic Pre-Conditioning) OR (Pre-Conditioning, Ischemic) OR (Preconditioning, Ischemic) OR (remote ischemic preconditioning) OR (remote ischaemic preconditioning) OR (remote preconditioning) OR (remote conditioning) OR (remote ischemic conditioning) OR (remote ischaemic conditioning) OR (transient limb ischemia) OR (muscle ischemia))

## S1.3.2 Web of Science

### Strategy 1. PAPE/PAP

**Records retrieved: 392**

TS=(Athletes OR Athlete OR "Professional Athletes" OR "Athlete, Professional" OR "Athletes, Professional" OR "Professional Athlete" OR "Elite Athletes" OR "Athlete, Elite" OR "Athletes, Elite" OR "Elite Athlete" OR "well-trained" OR "well trained")
AND
TS=("Athletic Performance" OR "Athletic Performances" OR "Performance, Athletic" OR "Performance, Sports" OR "Performances, Athletic" OR "Performances, Sports" OR "Sports Performance" OR "Sports Performances"OR sport* OR athletic* OR exercis*)
AND
TS=("post-activation potentiation" OR PAP OR "post activation performance enhancement" OR PAPE OR "conditioning contraction" OR "conditioning activity" OR "conditioning activities" OR "complex training")

### Strategy 2. Stretching and foam rolling

**Records retrieved: 350**

TS=(Athletes OR Athlete OR "Professional Athletes" OR "Athlete, Professional" OR "Athletes, Professional" OR "Professional Athlete" OR "Elite Athletes" OR "Athlete, Elite" OR "Athletes, Elite" OR "Elite Athlete" OR "well-trained" OR "well trained")
AND
TS=("Athletic Performance" OR "Athletic Performances" OR "Performance, Athletic" OR "Performance, Sports" OR "Performances, Athletic" OR "Performances, Sports" OR "Sports Performance" OR "Sports Performances"OR sport* OR athletic* OR exercis*)
AND
TS=(("static stretching" OR "static-stretching") OR ("dynamic stretching" OR "dynamic-stretching") OR ("ballistic stretching" OR "ballistic-stretching") OR ("proprioceptive neuromuscular facilitation" OR PNF OR "PNF-stretching") OR ("static-dynamic stretching" OR "static dynamic stretching") OR ("combined stretching" OR "combine of stretching" OR "stretching combination" OR "combined stretch*") OR ("foam rolling" OR "foam roll*" OR "foam roller" OR "self myofascial release" OR SMR) OR ("different stretching method*" OR "stretching method*"))

### Strategy 3. Passive heat maintenance

**Records retrieved: 32**

TS=(Athletes OR Athlete OR "Professional Athletes" OR "Athlete, Professional" OR "Athletes, Professional" OR "Professional Athlete" OR "Elite Athletes" OR "Athlete, Elite" OR "Athletes, Elite" OR "Elite Athlete" OR "well-trained" OR "well trained")
AND
TS=("Athletic Performance" OR "Athletic Performances" OR "Performance, Athletic" OR "Performance, Sports" OR "Performances, Athletic" OR "Performances, Sports" OR "Sports Performance" OR "Sports Performances"OR sport* OR athletic* OR exercis*)
AND
TS=("passive heat maintenance" OR "Passive Heat Maintenance" OR "passive heating" OR "passive heat" OR "heat maintenance" OR "heat-maintenance" OR "warm up maintenance" OR "warm-up maintenance" OR "warmup maintenance" OR "insulated clothing" OR "thermal clothing" OR "warming garment*" )

### Strategy 4. IPC/RIPC

**Records retrieved: 162**

TS=(Athletes OR Athlete OR "Professional Athletes" OR "Athlete, Professional" OR "Athletes, Professional" OR "Professional Athlete" OR "Elite Athletes" OR "Athlete, Elite" OR "Athletes, Elite" OR "Elite Athlete" OR "well-trained" OR "well trained")
AND
TS=("Athletic Performance" OR "Athletic Performances" OR "Performance, Athletic" OR "Performance, Sports" OR "Performances, Athletic" OR "Performances, Sports" OR "Sports Performance" OR "Sports Performances"OR sport* OR athletic* OR exercis*)
AND
TS=("ischemic preconditioning" OR "ischaemic preconditioning" OR "ischemic pre conditioning" OR "ischaemic pre conditioning" OR "ischemic pre-conditioning" OR "ischaemic pre-conditioning" OR preconditioning OR "remote ischemic preconditioning" OR "remote ischaemic preconditioning" OR "remote preconditioning" OR "remote conditioning" OR "remote ischemic conditioning" OR "remote ischaemic conditioning" OR "transient limb ischemia" OR "transient limb ischaemia" OR "muscle ischemia" OR "muscle ischaemia")

## S1.3.3 SPORTDiscus

### Strategy 1. PAPE/PAP

**Records retrieved: 119**

XB(athlete* OR "professional athlete*" OR "elite athlete*" OR "well-trained" OR "well trained")
AND
XB("athletic performance" OR "sports performance" OR "physical performance" OR performance OR sport* OR athletic* OR exercis*)
AND
XB("post-activation potentiation" OR "postactivation potentiation" OR PAP OR "post-activation performance enhancement" OR "postactivation performance enhancement" OR PAPE OR "conditioning contraction*" OR "conditioning activit*" OR "complex training")
AND
XB(random* OR randomized OR randomised OR trial OR "controlled trial" OR control* OR placebo OR sham OR crossover OR "cross-over")

### Strategy 2. Stretching and foam rolling

**Records retrieved: 182**

XB(athlete* OR "professional athlete*" OR "elite athlete*" OR "well-trained" OR "well trained")
AND
XB("athletic performance" OR "sports performance" OR "physical performance" OR performance OR sport* OR athletic* OR exercis*)
AND
XB("Static-Stretching" OR "Dynamic-Stretching" OR "Ballistic-Stretching"OR "Proprioceptive Neuromuscular Facilitation" OR "PNF-Stretching"OR "Static-Dynamic Stretching" OR "Combine of Stretching"OR "Foam Rolling" OR "Different stretching methods")
AND
XB(random* OR randomized OR randomised OR trial OR "controlled trial" OR control* OR placebo OR sham OR crossover OR "cross-over")

### Strategy 3. Passive heat maintenance

**Records retrieved: 6**

XB(athlete* OR "professional athlete*" OR "elite athlete*" OR "well-trained" OR "well trained")
AND
XB("athletic performance" OR "sports performance" OR "physical performance" OR performance OR sport* OR athletic* OR exercis*)
AND
XB("Passive Heat Maintenance" OR "passive heating" OR "heat maintenance" OR "insulated clothing" OR "warm up maintenance" OR "warm-up maintenance"OR "warmup maintenance" OR "thermal clothing" OR "warming garment*")
AND
XB(random* OR randomized OR randomised OR trial OR "controlled trial" OR control* OR placebo OR sham OR crossover OR "cross-over")

### Strategy 4. IPC/RIPC

**Records retrieved: 57**

XB(athlete* OR "professional athlete*" OR "elite athlete*" OR "well-trained" OR "well trained")
AND
XB("athletic performance" OR "sports performance" OR "physical performance" OR performance OR sport* OR athletic* OR exercis*)
AND
XB("ischemic preconditioning" OR "ischaemic preconditioning" OR "ischemic pre conditioning" OR "ischaemic pre conditioning" OR "ischemic pre-conditioning" OR "ischaemic pre-conditioning" OR preconditioning OR "remote ischemic preconditioning" OR "remote ischaemic preconditioning" OR "remote preconditioning" OR "remote conditioning" OR "remote ischemic conditioning" OR "remote ischaemic conditioning" OR "transient limb ischemia" OR "transient limb ischaemia" OR "muscle ischemia" OR "muscle ischaemia")
AND
XB(random* OR randomized OR randomised OR trial OR "controlled trial" OR control* OR placebo OR sham OR crossover OR "cross-over")

*Note: XB = SPORTDiscus expanded search field.*

## S1.3.4 CNKI

### Strategy 1. PAPE/PAP

**Records retrieved: 189**

SU=('运动员' + '竞技运动员' + '精英运动员' + '高水平运动员' + '专业运动员' + '优秀运动员')
AND
SU=('运动表现' + '运动成绩' + '竞技表现' + '体能表现' + '运动能力' + '运动绩效' + '竞技能力')
AND
TKA=('激活后增强' + '激活后增效' + '后激活增强' + '激活后增强效应' + '赛前激活' + '预激活'+ '复合训练' + '复合式训练' + '调节活动')

### Strategy 2. Stretching and foam rolling

**Records retrieved: 98**

SU=('运动员' + '竞技运动员' + '专业运动员' + '精英运动员' + '高水平运动员' + '优秀运动员' + '运动队')
AND
SU=('运动表现' + '运动成绩' + '竞技表现' + '体能表现' + '运动能力' + '运动绩效' + '竞技能力')
AND
TKA=('静态拉伸' + '动态拉伸' + '弹性拉伸' + 'PNF' + '本体感觉神经肌肉促进' + '神经肌肉促进法'+ '静态-动态拉伸' + '静态动态拉伸' + '组合拉伸' + '联合拉伸' + '拉伸组合' + '不同拉伸方法'+ '泡沫轴' + '泡沫滚压' + '滚泡沫轴' )

### Strategy 3. Passive heat maintenance

**Records retrieved: 3**

SU=('运动员' + '竞技运动员' + '专业运动员' + '精英运动员' + '高水平运动员' + '优秀运动员' )
AND
SU=('运动表现' + '运动成绩' + '竞技表现' + '体能表现' + '运动能力' + '运动绩效' + '竞技能力')
AND
TKA=('被动加热' + '被动热身' + '被动升温' + '热维持' + '保温维持' + '热量维持'+ '热身维持' + '热身后维持' + '热身维持策略'+ '保温服' + '保温衣' + '保暖衣' + '保暖服' + '保暖装备'+ '隔热服' + '隔热衣' + '热防护服' + '被动热维持' + '热维持策略')

### Strategy 4. IPC/RIPC

**Records retrieved: 15**

SU=('运动员' + '竞技运动员' + '专业运动员' + '精英运动员' + '高水平运动员' + '优秀运动员' )
AND
SU=('运动表现' + '运动成绩' + '竞技表现' + '体能表现' + '运动能力' + '运动绩效' + '竞技能力')
AND
TKA=('缺血预处理' + '缺血预适应' + '缺血预处理策略' + '缺血预调节'+ '远隔缺血预处理' + '远隔缺血预适应' + '远程缺血预处理'+ '远隔缺血调节' + '远程缺血调节' + '远隔缺血预调节')

*Note: SU = subject field; TKA = title/keyword/abstract field.*

## S1.3.5 Scopus

### Strategy 1. PAPE/PAP

**Records retrieved: 187**

TITLE-ABS-KEY(athlete* OR "professional athlete*" OR "elite athlete*" OR "well-trained" OR "well trained")
AND
TITLE-ABS-KEY("athletic performance" OR "athletic performances" OR "performance, athletic" OR "performance, sports" OR "performances, athletic" OR "performances, sports" OR "sport performance" OR "sports performance" OR "sports performances" OR sport* OR athletic* OR exercis*)
AND
TITLE-ABS-KEY("post-activation potentiation" OR "postactivation potentiation" OR PAP OR "post-activation performance enhancement" OR "postactivation performance enhancement" OR PAPE OR "conditioning contraction*" OR "conditioning activit*" OR "complex training")

### Strategy 2. Stretching and foam rolling

**Records retrieved: 179**

TITLE-ABS-KEY(athlete* OR "professional athlete*" OR "elite athlete*" OR "well-trained" OR "well trained")
AND
TITLE-ABS-KEY("athletic performance" OR "athletic performances" OR "performance, athletic" OR "performance, sports" OR "performances, athletic" OR "performances, sports" OR "sport performance" OR "sports performance" OR "sports performances" OR sport* OR athletic* OR exercis*)
AND
TITLE-ABS-KEY("static stretching" OR "static-stretching" OR "dynamic stretching" OR "dynamic-stretching" OR "ballistic stretching" OR "ballistic-stretching" OR "proprioceptive neuromuscular facilitation" OR PNF OR "PNF stretching" OR "PNF-stretching" OR "static-dynamic stretching" OR "static dynamic stretching" OR "combined stretching" OR "combine of stretching" OR "stretching combination" OR "combined stretch*" OR "foam rolling" OR "foam roll*" OR "foam roller*" OR "self-myofascial release" OR "self myofascial release" OR SMR OR "different stretching method*" OR "stretching method*")

### Strategy 3. Passive heat maintenance

**Records retrieved: 14**

TITLE-ABS-KEY(athlete* OR "professional athlete*" OR "elite athlete*" OR "well-trained" OR "well trained")
AND
TITLE-ABS-KEY("athletic performance" OR "athletic performances" OR "performance, athletic" OR "performance, sports" OR "performances, athletic" OR "performances, sports" OR "sport performance" OR "sports performance" OR "sports performances" OR sport* OR athletic* OR exercis*)
AND
TITLE-ABS-KEY("passive heat maintenance" OR "passive heating" OR "passive heat" OR "heat maintenance" OR "heat-maintenance" OR "warm up maintenance" OR "warm-up maintenance" OR "warmup maintenance" OR "insulated clothing" OR "thermal clothing" OR "warming garment*")

### Strategy 4. IPC/RIPC

**Records retrieved: 90**

TITLE-ABS-KEY(athlete* OR "professional athlete*" OR "elite athlete*" OR "well-trained" OR "well trained")
AND
TITLE-ABS-KEY("athletic performance" OR "athletic performances" OR "performance, athletic" OR "performance, sports" OR "performances, athletic" OR "performances, sports" OR "sport performance" OR "sports performance" OR "sports performances" OR sport* OR athletic* OR exercis*)
AND
TITLE-ABS-KEY("ischemic preconditioning" OR "ischaemic preconditioning" OR "ischemic pre conditioning" OR "ischaemic pre conditioning" OR "ischemic pre-conditioning" OR "ischaemic pre-conditioning" OR preconditioning OR "remote ischemic preconditioning" OR "remote ischaemic preconditioning" OR "remote preconditioning" OR "remote conditioning" OR "remote ischemic conditioning" OR "remote ischaemic conditioning" OR "transient limb ischemia" OR "transient limb ischaemia" OR "muscle ischemia" OR "muscle ischaemia")

*Note: TITLE-ABS-KEY = article title, abstract, and keywords fields in Scopus.*

## S1.3.6 Embase

### Strategy 1. PAPE/PAP

**Records retrieved: 113**

('athlete'/exp OR athlete*:ti,ab,kw OR 'professional athlete':ti,ab,kw OR 'professional athletes':ti,ab,kw OR 'elite athlete':ti,ab,kw OR 'elite athletes':ti,ab,kw OR 'well-trained':ti,ab,kw OR 'well trained':ti,ab,kw)
AND
('athletic performance'/exp OR 'athletic performance':ti,ab,kw OR 'athletic performances':ti,ab,kw OR 'sport performance':ti,ab,kw OR 'sports performance':ti,ab,kw OR 'sports performances':ti,ab,kw OR sport*:ti,ab,kw OR athletic*:ti,ab,kw OR exercis*:ti,ab,kw)
AND
('post-activation potentiation':ti,ab,kw OR 'postactivation potentiation':ti,ab,kw OR PAP:ti,ab,kw OR 'post-activation performance enhancement':ti,ab,kw OR 'postactivation performance enhancement':ti,ab,kw OR PAPE:ti,ab,kw OR 'conditioning contraction':ti,ab,kw OR 'conditioning contractions':ti,ab,kw OR 'conditioning activity':ti,ab,kw OR 'conditioning activities':ti,ab,kw OR 'complex training':ti,ab,kw)

### Strategy 2. Stretching and foam rolling

**Records retrieved: 270**

('athlete'/exp OR athlete*:ti,ab,kw OR 'professional athlete':ti,ab,kw OR 'professional athletes':ti,ab,kw OR 'elite athlete':ti,ab,kw OR 'elite athletes':ti,ab,kw OR 'well-trained':ti,ab,kw OR 'well trained':ti,ab,kw)
AND
('athletic performance'/exp OR 'athletic performance':ti,ab,kw OR 'athletic performances':ti,ab,kw OR 'sport performance':ti,ab,kw OR 'sports performance':ti,ab,kw OR 'sports performances':ti,ab,kw OR sport*:ti,ab,kw OR athletic*:ti,ab,kw OR exercis*:ti,ab,kw)
AND
('stretching exercise'/exp OR 'static stretching':ti,ab,kw OR 'static-stretching':ti,ab,kw OR 'dynamic stretching':ti,ab,kw OR 'dynamic-stretching':ti,ab,kw OR 'ballistic stretching':ti,ab,kw OR 'ballistic-stretching':ti,ab,kw OR 'proprioceptive neuromuscular facilitation':ti,ab,kw OR PNF:ti,ab,kw OR 'PNF stretching':ti,ab,kw OR 'PNF-stretching':ti,ab,kw OR 'static-dynamic stretching':ti,ab,kw OR 'static dynamic stretching':ti,ab,kw OR 'combined stretching':ti,ab,kw OR 'combine of stretching':ti,ab,kw OR 'stretching combination':ti,ab,kw OR 'foam rolling':ti,ab,kw OR 'foam roll':ti,ab,kw OR 'foam rolls':ti,ab,kw OR 'foam roller':ti,ab,kw OR 'foam rollers':ti,ab,kw OR 'self-myofascial release':ti,ab,kw OR 'self myofascial release':ti,ab,kw OR SMR:ti,ab,kw OR 'different stretching methods':ti,ab,kw OR 'stretching methods':ti,ab,kw)

### Strategy 3. Passive heat maintenance

**Records retrieved: 8**

('athlete'/exp OR athlete*:ti,ab,kw OR 'professional athlete':ti,ab,kw OR 'professional athletes':ti,ab,kw OR 'elite athlete':ti,ab,kw OR 'elite athletes':ti,ab,kw OR 'well-trained':ti,ab,kw OR 'well trained':ti,ab,kw)
AND
('athletic performance'/exp OR 'athletic performance':ti,ab,kw OR 'athletic performances':ti,ab,kw OR 'sport performance':ti,ab,kw OR 'sports performance':ti,ab,kw OR 'sports performances':ti,ab,kw OR sport*:ti,ab,kw OR athletic*:ti,ab,kw OR exercis*:ti,ab,kw)
AND
('passive heat maintenance':ti,ab,kw OR 'passive heating':ti,ab,kw OR 'passive heat':ti,ab,kw OR 'heat maintenance':ti,ab,kw OR 'heat-maintenance':ti,ab,kw OR 'warm up maintenance':ti,ab,kw OR 'warm-up maintenance':ti,ab,kw OR 'warmup maintenance':ti,ab,kw OR 'insulated clothing':ti,ab,kw OR 'thermal clothing':ti,ab,kw OR 'warming garment':ti,ab,kw OR 'warming garments':ti,ab,kw)

### Strategy 4. IPC/RIPC

**Records retrieved: 60**

('athlete'/exp OR athlete*:ti,ab,kw OR 'professional athlete':ti,ab,kw OR 'professional athletes':ti,ab,kw OR 'elite athlete':ti,ab,kw OR 'elite athletes':ti,ab,kw OR 'well-trained':ti,ab,kw OR 'well trained':ti,ab,kw)
AND
('athletic performance'/exp OR 'athletic performance':ti,ab,kw OR 'athletic performances':ti,ab,kw OR 'sport performance':ti,ab,kw OR 'sports performance':ti,ab,kw OR 'sports performances':ti,ab,kw OR sport*:ti,ab,kw OR athletic*:ti,ab,kw OR exercis*:ti,ab,kw)
AND
('ischemic preconditioning'/exp OR 'ischemic preconditioning':ti,ab,kw OR 'ischaemic preconditioning':ti,ab,kw OR 'ischemic pre conditioning':ti,ab,kw OR 'ischaemic pre conditioning':ti,ab,kw OR 'ischemic pre-conditioning':ti,ab,kw OR 'ischaemic pre-conditioning':ti,ab,kw OR preconditioning:ti,ab,kw OR 'remote ischemic preconditioning':ti,ab,kw OR 'remote ischaemic preconditioning':ti,ab,kw OR 'remote preconditioning':ti,ab,kw OR 'remote conditioning':ti,ab,kw OR 'remote ischemic conditioning':ti,ab,kw OR 'remote ischaemic conditioning':ti,ab,kw OR 'transient limb ischemia':ti,ab,kw OR 'transient limb ischaemia':ti,ab,kw OR 'muscle ischemia':ti,ab,kw OR 'muscle ischaemia':ti,ab,kw)

*Note: The Embase strategies use Embase.com command-line syntax. /exp indicates explosion of an Emtree term; ti, ab, and kw indicate title, abstract, and author-keyword fields, respectively.*

# Supplementary Section S2. Risk-of-Bias Judgments

**Supplementary Figure S1A–D. Study-level risk-of-bias judgments for the 90 independent datasets.**

*Note: Panels A–D present the 90 independent datasets in the order reported in Supplementary Table 1. D1, random sequence generation; D2, allocation concealment; D3, blinding of participants and personnel; D4, blinding of outcome assessment; D5, incomplete outcome data; D6, selective reporting; D7, other bias. Green indicates low risk of bias, yellow indicates unclear risk of bias, and red indicates high risk of bias.*

# Supplementary Section S3. Additional Network Meta-Analysis Diagnostics

## S3.1 Between-study heterogeneity

**Supplementary Table S2. Between-study heterogeneity estimates for the outcome-specific networks**

| **Outcome network** | **Datasets, k** | **τ² (95% CI)** | **Interpretation** |
| --- | --- | --- | --- |
| Explosive/jump performance | 42 | 0.014 (0.001–0.394) | Low estimated heterogeneity; wide CI |
| Speed/change-of-direction performance | 36 | <0.001; CI not estimable | Near-zero boundary estimate |
| Sport-specific performance | 22 | <0.001; CI not estimable | Near-zero boundary estimate |
| Endurance performance | 14 | <0.001; CI not estimable | Near-zero boundary estimate |

*Note: CI, confidence interval. Confidence intervals were not estimable when τ² was located at the boundary of zero.*

## S3.2 Small-study effects

**Supplementary Table S3. Results of Egger’s regression tests for small-study effects**

| **Outcome network** | **Intercept** | **Cluster-robust SE** | **P value** |
| --- | --- | --- | --- |
| Explosive/jump performance | -0.319 | 0.834 | 0.704 |
| Speed/change-of-direction performance | -1.118 | 0.660 | 0.100 |
| Sport-specific performance | 0.690 | 1.167 | 0.561 |
| Endurance performance | 1.058 | 0.778 | 0.197 |

*Note: Cluster-robust standard errors were used to account for multiple effect estimates contributed by the same study. A two-sided P value < 0.05 was considered indicative of possible small-study effects.*

# Supplementary Section S4. CINeMA Confidence Assessments

Confidence in the network estimates was assessed using CINeMA across six domains: within-study bias, reporting bias, indirectness, imprecision, heterogeneity, and incoherence. A value of 0 in the “Number of direct studies” column indicates that the comparison was informed entirely by indirect evidence.

## S4.1 Explosive/jump performance

**Supplementary Table S4. CINeMA assessment of confidence in network estimates for explosive/jump performance.**

| **Comparison** | **Number of direct studies** | **Within-study bias** | **Reporting bias** | **Indirectness** | **Imprecision** | **Heterogeneity** | **Incoherence** | **Confidence rating** |
| --- | --- | --- | --- | --- | --- | --- | --- | --- |
| DS:SS | 7 | No concerns | Low risk | No concerns | No concerns | No concerns | No concerns | High |
| DS:WU | 5 | No concerns | Low risk | No concerns | Some concerns | No concerns | No concerns | Moderate |
| DS-SS:SS | 1 | No concerns | Low risk | No concerns | Major concerns | No concerns | No concerns | Low |
| DS-SS:WU | 1 | No concerns | Low risk | No concerns | Major concerns | No concerns | No concerns | Low |
| FR:SS | 1 | No concerns | Low risk | No concerns | No concerns | No concerns | No concerns | High |
| FR:WU | 2 | No concerns | Low risk | No concerns | Some concerns | No concerns | No concerns | Moderate |
| HEAT:WU | 2 | No concerns | Low risk | No concerns | Some concerns | No concerns | No concerns | Moderate |
| IPC:WU | 2 | No concerns | Low risk | Some concerns | Major concerns | No concerns | No concerns | Very low |
| PAPE:SS | 1 | No concerns | Low risk | No concerns | No concerns | No concerns | No concerns | High |
| PAPE:WU | 16 | No concerns | Low risk | No concerns | No concerns | No concerns | No concerns | High |
| SHAM:WU | 1 | No concerns | Low risk | Some concerns | Major concerns | No concerns | No concerns | Very low |
| SS:SS-DS | 4 | No concerns | Low risk | No concerns | Some concerns | No concerns | No concerns | Moderate |
| SS:WU | 8 | No concerns | Low risk | No concerns | No concerns | Some concerns | No concerns | Moderate |
| SS-DS:WU | 2 | No concerns | Low risk | No concerns | Major concerns | No concerns | No concerns | Low |
| DS-FR:SS | 0 | No concerns | Low risk | No concerns | Major concerns | No concerns | No concerns | Low |
| DS-FR:WU | 0 | No concerns | Low risk | No concerns | Major concerns | No concerns | No concerns | Low |
| HEAT:SS | 0 | No concerns | Low risk | No concerns | No concerns | No concerns | No concerns | High |
| IPC:SS | 0 | No concerns | Low risk | No concerns | Some concerns | No concerns | No concerns | Moderate |
| SHAM:SS | 0 | No concerns | Low risk | No concerns | Major concerns | No concerns | No concerns | Low |

## S4.2 Speed/change-of-direction performance

**Supplementary Table S5. CINeMA assessment of confidence in network estimates for speed/change-of-direction performance.**

| **Comparison** | **Number of direct studies** | **Within-study bias** | **Reporting bias** | **Indirectness** | **Imprecision** | **Heterogeneity** | **Incoherence** | **Confidence rating** |
| --- | --- | --- | --- | --- | --- | --- | --- | --- |
| DS:SS | 7 | No concerns | Low risk | No concerns | No concerns | No concerns | No concerns | High |
| DS:WU | 5 | No concerns | Low risk | No concerns | No concerns | No concerns | No concerns | High |
| DS-SS:SS | 2 | No concerns | Low risk | No concerns | Major concerns | No concerns | No concerns | Low |
| DS-SS:WU | 2 | No concerns | Low risk | No concerns | Major concerns | No concerns | No concerns | Low |
| FR:WU | 1 | No concerns | Low risk | No concerns | No concerns | Some concerns | No concerns | Moderate |
| HEAT:WU | 4 | No concerns | Low risk | No concerns | Some concerns | No concerns | No concerns | Moderate |
| IPC:WU | 1 | No concerns | Low risk | Some concerns | Major concerns | No concerns | No concerns | Very low |
| PAPE:WU | 18 | No concerns | Low risk | No concerns | No concerns | No concerns | No concerns | High |
| SHAM:WU | 1 | No concerns | Low risk | Some concerns | Major concerns | No concerns | No concerns | Very low |
| SS:SS-DS | 4 | No concerns | Low risk | Some concerns | No concerns | No concerns | No concerns | Moderate |
| SS:WU | 7 | No concerns | Low risk | No concerns | Some concerns | No concerns | No concerns | Moderate |
| SS-DS:WU | 3 | No concerns | Low risk | No concerns | Some concerns | No concerns | No concerns | Moderate |
| DS-FR:SS | 0 | No concerns | Low risk | No concerns | Major concerns | No concerns | No concerns | Low |
| DS-FR:WU | 0 | No concerns | Low risk | No concerns | Major concerns | No concerns | No concerns | Low |
| FR:SS | 0 | No concerns | Low risk | No concerns | No concerns | No concerns | No concerns | High |
| HEAT:SS | 0 | No concerns | Low risk | No concerns | No concerns | No concerns | No concerns | High |
| IPC:SS | 0 | No concerns | Low risk | Some concerns | Major concerns | No concerns | No concerns | Very low |
| PAPE:SS | 0 | No concerns | Low risk | No concerns | No concerns | No concerns | No concerns | High |
| RIPC:SS | 0 | No concerns | Low risk | No concerns | Major concerns | No concerns | No concerns | Low |
| RIPC:WU | 0 | No concerns | Low risk | Some concerns | Major concerns | No concerns | No concerns | Very low |
| SHAM:SS | 0 | No concerns | Low risk | Some concerns | Major concerns | No concerns | No concerns | Very low |

## S4.3 Sport-specific performance

**Supplementary Table S6. CINeMA assessment of confidence in network estimates for sport-specific performance.**

| **Comparison** | **Number of direct studies** | **Within-study bias** | **Reporting bias** | **Indirectness** | **Imprecision** | **Heterogeneity** | **Incoherence** | **Confidence rating** |
| --- | --- | --- | --- | --- | --- | --- | --- | --- |
| DS:WU | 1 | No concerns | Low risk | Some concerns | Major concerns | No concerns | No concerns | Very low |
| FR:WU | 1 | No concerns | Low risk | No concerns | Major concerns | No concerns | No concerns | Low |
| HEAT:WU | 2 | No concerns | Low risk | No concerns | Major concerns | No concerns | No concerns | Low |
| IPC:SHAM | 4 | No concerns | Low risk | No concerns | No concerns | No concerns | No concerns | High |
| IPC:WU | 1 | No concerns | Low risk | Some concerns | Major concerns | No concerns | No concerns | Very low |
| PAPE:WU | 12 | No concerns | Low risk | No concerns | No concerns | No concerns | No concerns | High |
| DS:SHAM | 0 | No concerns | Low risk | Some concerns | Major concerns | No concerns | No concerns | Very low |
| FR:SHAM | 0 | No concerns | Low risk | Some concerns | Major concerns | No concerns | No concerns | Very low |
| HEAT:SHAM | 0 | No concerns | Low risk | Some concerns | Major concerns | No concerns | No concerns | Very low |
| PAPE:SHAM | 0 | No concerns | Low risk | Some concerns | Major concerns | No concerns | No concerns | Very low |
| SHAM:SS | 0 | No concerns | Low risk | Some concerns | Major concerns | No concerns | No concerns | Very low |
| SHAM:SS-DS | 0 | No concerns | Low risk | Some concerns | Major concerns | No concerns | No concerns | Very low |
| SHAM:WU | 0 | No concerns | Low risk | Some concerns | Major concerns | No concerns | No concerns | Very low |
| SS:WU | 0 | No concerns | Low risk | No concerns | Major concerns | No concerns | No concerns | Low |
| SS-DS:WU | 0 | No concerns | Low risk | No concerns | Major concerns | No concerns | No concerns | Low |

## S4.4 Endurance performance

**Supplementary Table S7. CINeMA assessment of confidence in network estimates for endurance performance.**

| **Comparison** | **Number of direct studies** | **Within-study bias** | **Reporting bias** | **Indirectness** | **Imprecision** | **Heterogeneity** | **Incoherence** | **Confidence rating** |
| --- | --- | --- | --- | --- | --- | --- | --- | --- |
| IPC:WU | 1 | No concerns | Low risk | Some concerns | Some concerns | No concerns | No concerns | Low |
| PAPE:WU | 2 | No concerns | Low risk | No concerns | Major concerns | No concerns | No concerns | Low |
| RIPC:WU | 2 | No concerns | Low risk | No concerns | No concerns | No concerns | No concerns | High |
| SHAM:WU | 1 | No concerns | Low risk | No concerns | Some concerns | Some concerns | No concerns | Low |
| SS:WU | 3 | No concerns | Low risk | No concerns | Major concerns | No concerns | No concerns | Low |
| IPC:PAPE | 0 | No concerns | Low risk | No concerns | Some concerns | Some concerns | No concerns | Low |
| PAPE:RIPC | 0 | No concerns | Low risk | No concerns | Some concerns | No concerns | No concerns | Moderate |
| PAPE:SHAM | 0 | No concerns | Low risk | No concerns | No concerns | Some concerns | No concerns | Moderate |
| PAPE:SS | 0 | No concerns | Low risk | No concerns | Major concerns | No concerns | No concerns | Low |
| RIPC:SS | 0 | No concerns | Low risk | No concerns | Major concerns | No concerns | No concerns | Low |
| SHAM:SS | 0 | No concerns | Low risk | No concerns | No concerns | No concerns | No concerns | High |

# Supplementary Section S5. Sensitivity Analyses

Sensitivity analyses examined whether alternative assumptions regarding within-participant correlations affected the magnitude, statistical significance, or interpretation of the main network estimates and SUCRA rankings.

## S5.1 Assumed within-participant correlations

**Supplementary Table S8. Assumptions used for effective sample-size adjustment in sensitivity analyses.**

| **Scenario** | **Assumed within-participant correlation** | **Adjusted sample size** |
| --- | --- | --- |
| Sensitivity 1 | r = 0.30 | n / 0.70 |
| Sensitivity 2 | r = 0.50 | n / 0.50 |
| Sensitivity 3 | r = 0.70 | n / 0.30 |

## S5.2 Sensitivity analyses of key network estimates

**Supplementary Table S9. Sensitivity analyses of key network comparisons under assumed within-participant correlation coefficients of 0.30, 0.50, and 0.70.**

| **Outcome** | **Main comparison** | **Main analysis SMD [95% CI]** | **r = 0.30** | **r = 0.50** | **r = 0.70** | **Interpretation changed?** |
| --- | --- | --- | --- | --- | --- | --- |
| Explosive/jump | PAPE vs WU | 0.40 (0.20, 0.59) | 0.41 (0.22, 0.60) | 0.41 (0.23, 0.60) | 0.42 (0.24, 0.60) | No |
| Explosive/jump | PAPE vs SS | 0.60 (0.32, 0.88) | 0.59 (0.32, 0.87) | 0.59 (0.32, 0.86) | 0.59 (0.33, 0.85) | No |
| Explosive/jump | DS vs SS | 0.38 (0.15, 0.60) | 0.38 (0.16, 0.61) | 0.39 (0.17, 0.61) | 0.39 (0.17, 0.61) | No |
| Explosive/jump | FR vs SS | 0.36 (0.05, 0.68) | 0.36 (0.04, 0.68) | 0.36 (0.04, 0.68) | 0.36 (0.04, 0.69) | No |
| Speed/COD | FR vs WU | 0.66 (0.10, 1.21) | 0.67 (0.18, 1.17) | 0.69 (0.21, 1.18) | 0.72 (0.24, 1.20) | No |
| Speed/COD | PAPE vs WU | 0.31 (0.14, 0.48) | 0.31 (0.15, 0.46) | 0.31 (0.16, 0.47) | 0.32 (0.17, 0.48) | No |
| Speed/COD | DS vs WU | 0.32 (0.06, 0.57) | 0.33 (0.10, 0.56) | 0.35 (0.12, 0.58) | 0.37 (0.14, 0.61) | No |
| Speed/COD | FR vs SS | 0.83 (0.25, 1.41) | 0.85 (0.33, 1.37) | 0.88 (0.37, 1.39) | 0.92 (0.41, 1.43) | No |
| Speed/COD | PAPE vs SS | 0.48 (0.20, 0.77) | 0.49 (0.23, 0.75) | 0.50 (0.24, 0.77) | 0.52 (0.26, 0.79) | No |
| Speed/COD | HEAT vs SS | 0.49 (0.09, 0.88) | 0.49 (0.14, 0.85) | 0.51 (0.15, 0.86) | 0.52 (0.16, 0.88) | No |
| Speed/COD | DS vs SS | 0.49 (0.25, 0.73) | 0.51 (0.28, 0.73) | 0.54 (0.31, 0.77) | 0.57 (0.34, 0.80) | No |
| Speed/COD | SS-DS vs SS | 0.31 (0.02, 0.60) | 0.32 (0.05, 0.59) | 0.33 (0.05, 0.60) | 0.34 (0.05, 0.62) | No |
| Sport-specific | IPC vs SHAM | 0.54 (0.16, 0.92) | 0.54 (0.21, 0.87) | 0.54 (0.21, 0.86) | 0.53 (0.21, 0.86) | No |
| Sport-specific | PAPE vs WU | 0.31 (0.10, 0.52) | 0.31 (0.13, 0.50) | 0.32 (0.14, 0.50) | 0.32 (0.14, 0.51) | No |
| Endurance | RIPC vs WU | 0.62 (0.20, 1.05) | 0.62 (0.27, 0.98) | 0.63 (0.32, 0.93) | 0.62 (0.32, 0.93) | No |
| Endurance | RIPC vs PAPE | 0.73 (0.06, 1.39) | 0.73 (0.17, 1.29) | 0.73 (0.26, 1.21) | 0.74 (0.27, 1.21) | No |
| Endurance | RIPC vs SS | 0.81 (0.16, 1.46) | 0.82 (0.27, 1.36) | 0.82 (0.36, 1.28) | 0.82 (0.38, 1.26) | No |

## S5.3 Stability of intervention rankings

**Supplementary Table S10. Stability of SUCRA-based top-three rankings across sensitivity analyses.**

| **Outcome** | **Main analysis top 3** | **r = 0.30 top 3** | **r = 0.50 top 3** | **r = 0.70 top 3** | **Ranking stable?** |
| --- | --- | --- | --- | --- | --- |
| Explosive/jump | PAPE, HEAT, DS | PAPE, HEAT, DS | PAPE, HEAT, DS | PAPE, HEAT, DS | Yes |
| Speed/COD | FR, DS, PAPE | FR, DS, HEAT | FR, DS, PAPE | FR, DS, PAPE | Mostly stable |
| Sport-specific | IPC, PAPE, DS | IPC, PAPE, DS | IPC, PAPE, DS | IPC, PAPE, DS | Yes |
| Endurance | RIPC, IPC, SHAM | RIPC, IPC, SHAM | RIPC, IPC, SHAM | RIPC, IPC, SHAM | Yes |

# Supplementary Section S6. Abbreviations

| **Abbreviation** | **Definition** |
| --- | --- |
| **CI** | Confidence interval |
| **CINeMA** | Confidence in Network Meta-Analysis |
| **CNKI** | China National Knowledge Infrastructure |
| **COD** | Change of direction |
| **DS** | Dynamic stretching |
| **DS-FR** | Dynamic stretching combined with foam rolling |
| **DS-SS** | Dynamic stretching followed by static stretching |
| **FR** | Foam rolling |
| **HEAT** | Passive heat maintenance |
| **IPC** | Ischemic preconditioning |
| **PAPE** | Post-activation performance enhancement |
| **RIPC** | Remote ischemic preconditioning |
| **SHAM** | Sham stimulation |
| **SMD** | Standardized mean difference |
| **SS** | Static stretching |
| **SS-DS** | Static stretching followed by dynamic stretching |
| **SUCRA** | Surface under the cumulative ranking curve |
| **WU** | Regular warm-up |
