# Supplementary material for: Effects of pre-competition conditioning strategies on acute sport performance in high-level athletes: a systematic review and network meta-analysis
Source: Front Physiol. 2026 Oct 1;17:1930250. doi: 10.3389/fphys.2026.1930250 (PMC13669563; doi:10.3389/fphys.2026.1930250)
Supplement: Supplementary file 2 [file Table1.docx]

Supplementary table 1. Characteristics of the included studies.

| **Author, year** | **Country** | **Sport** | **Training and performance caliber** | **Sample size (N; M/F)** | **Age** | **Intervention/comparator** | **Key intervention parameters** | **Intervention-to-test interval** | **Outcome category** | **Main outcome** |
| --- | --- | --- | --- | --- | --- | --- | --- | --- | --- | --- |
| Aandahl et al. (2018) | Norway | Taekwondo, kickboxing | 2 | 16; 11/5 | 20.6±5.5 | WU vs PAPE | PAPE; elastic-band kicking; 3 × 10 repetitions; initial tension 30 N and approximately 60 N; 90 s between sets | 5–8 min | ① | Linear foot velocity during roundhouse kick |
| Albuquerque et al. (2024) | Brazil | Taekwondo | 3 | 11; 7/4 | 19.0±3.0 | IPC vs SHAM | IPC; bilateral legs; 4 × (5 + 5 min); IOP + 20 mmHg | 0 min | ②, ③ | CMJ; time to exhaustion in the PSTT |
| Andrić et al. (2026) | Serbia | Team sports | 3 | 10; 7/3 | 28.0±9.0 | WU vs PAPE | PAPE; functional eccentric-overload flywheel half-squat; 3 × 6 repetitions; inertia 0.025 kg·m²; 2 min between sets | 0 min | ② | CMJ |
| Aydın et al. (2026) | Türkiye | Judo | 3 | 20; 20/0 | 19.6±1.0 | PAPE vs WU | PAPE; back squat; 1 × 6 repetitions; 80% 6RM. | 7 min | ① | Special Judo Fitness Test index |
| Aytac et al. (2024) | Türkiye | Team sports | 3 | 18; 18/0 | 23.6±3.3 | WU vs PAPE | PAPE; back squat; 3 × 3 repetitions; 90% 1RM | 8 min | ④ | 505 test |
| Baiget et al. (2023) | Spain | Tennis | 2 | 16; 8/8 | 16.9±1.1 | WU vs PAPE | PAPE; shoulder internal rotation and shoulder flexion MVIC; 1 × 5 s each | 0 min | ① | Serve velocity |
| Barbosa et al. (2020) | Singapore | Swimming | 2 | 12; 12/0 | 23.50±3.35 | WU vs PAPE | PAPE; resistance-band exercise; 2 × 5 repetitions; maximal intensity; 2 min between sets | 8 min | ① | 25 m maximal freestyle swimming velocity |
| Bayazit and Turna (2022) | Türkiye | Archery | 4 | 13; 13/0 | 17.23±1.87 | WU vs PAPE | PAPE; bent-over row; 1 × 3 repetitions; 85% 1RM | 5 min | ① | 60 m shooting score test |
| Beaven et al. (2018) | New Zealand | Rugby sevens | 2 | 12; 12/0 | 21.5±1.3 | WU vs HEAT | HEAT; insulated garment; 15 min | 0 min | ④ | 5 × 40 m repeated sprint |
| Blagrove et al. (2019) | UK | Long-distance running | 3 | 17; 17/0 | 17.6±1.2 | WU vs PAPE | PAPE; drop jump; 6 repetitions | 10 min | ③ | time to exhaustion |
| Brink et al. (2022a) | South Africa | Football | 2 | 69; 69/0 | 24.0±5.0 | WU vs PAPE | PAPE; 20 m maximal sprint; 2 repetitions; ≥95% baseline speed | 6 min | ④ | 10 m sprint velocity |
| Brink et al. (2022b) | South Africa | Football | 2 | 69; 69/0 | 24.0±5.0 | WU vs PAPE | PAPE; maximal vertical jump; 2 repetitions; ≥95% baseline peak value | 2 min | ② | CMJ |
| Çabuk and İnce (2025) | Türkiye | Football | 2 | 40; 40/0 | 15.57±0.81 | WU vs PAPE | PAPE; hip thrust; 1 × 6 repetitions; 84% 1RM | 7 min | ②, ④ | 10 m sprint time; relative peak power |
| Ceylan and Franchini (2022) | Türkiye, Brazil | Judo | 4 | 10; 10/0 | 20.0±2.2 | WU vs IPC | IPC; proximal right leg; 3 × (5 + 5 min); 220 mmHg | 0 min | ① | Special Judo Fitness Test index |
| Chaouachi et al. (2010) | Tunisia | Mixed sports: athletics, rugby, football, gymnastics, and combat sports | 4 | 19; 19/0 | 20.6±1.2 | WU vs SS vs DS vs SS-DS vs DS-SS | SS; five lower-limb muscle groups; bilateral 2 × 30 s; 10 s between exercises  DS; five lower-limb muscle groups; bilateral 2 × 30 s; 10 s between exercises  SS-DS; SS followed by DS; 1 × 30 s per muscle group for each modality  DS-SS; DS followed by SS; 1 × 30 s per muscle group for each modality | 2 min | ②, ④ | CMJ; 10 m sprint time |
| Chen et al. (2023) | China | Long-distance running | 2 | 16; 16/0 | 19.9±1.3 | WU vs IPC vs RIPC | IPC; proximal bilateral legs; 4 × (5 + 5 min); 220 mmHg.  RIPC; proximal bilateral upper limbs; 4 × (5 + 5 min); 220 mmHg | 30 min | ③ | Maximal accumulated oxygen deficit |
| Chua et al. (2021) | Singapore | Badminton | 3 | 14; 11/3 | 19.0±2.3 | WU vs PAPE | PAPE; sport-specific potentiation exercises; weighted vest and wrist weights | 0 min | ①, ④ | Spike velocity; change-of-direction speed |
| Cocking et al. (2018) | UK, Qatar | Cycling | 2 | 12; NR | 36.0±7.0 | SHAM vs RIPC vs IPC | IPC; bilateral legs; 4 × (5 + 5 min); 220 mmHg  RIPC; bilateral upper limbs; 4 × (5 + 5 min); 220 mmHg | 20 min | ③ | Completion time in a 375 kJ time trial |
| Cuenca-Fernández et al. (2026) | Spain | Swimming | 4 | 14; 7/7 | 19.4±2.21 | WU vs SS vs PAPE | SS; latissimus dorsi and pectoralis major; 2 × 30 s per muscle group  PAPE; elastic-band straight-arm pulldown; 3 × 5 repetitions; maximal effort | 5 min | ② | Power output |
| de Oliveira and Rama (2016) | Portugal | Mixed sports: handball, rugby | 2 | 22; 22/0 | 23.2±5.0 | WU vs SS | SS; five exercises; 30 s bilaterally; total duration 5 min | 0 min | ②, ④ | CMJ; 20 m sprint velocity |
| de Paula Simola et al. (2026) | Brazil | Mountain biking | 2 | 10; 10/0 | 32.0±8.0 | WU vs PAPE | PAPE; isometric half-squat combined with mechanical vibration; 1 × 10 s; 26 Hz | 11 min | ② | Peak power |
| Dello Iacono et al. (2016) | Israel | Handball, basketball | 4 | 26; 26/0 | 15.4±0.3 | WU vs PAPE | PAPE; bilateral depth jumps; 3 × 10 repetitions | 8 min | ②, ④ | CMJ; 20 m sprint time |
| Di Cagno et al. (2010) | Italy | Rhythmic gymnastics | 4 | 38; 0/38 | 14.1±3.2 | WU vs SS | SS; five lower-limb muscle groups; bilateral 2 × 30 s; 10 s between exercises | 1 min | ② | Flight time during sport-specific jumps |
| dos Santos Silva et al. (2021) | Brazil | Long jump | 2 | 11; NR | 16.2±1.3 | WU vs PAPE | PAPE; consecutive single-leg vertical jumps; 4 repetitions per leg | 3 min | ② | Flight height during long jump |
| Eyuboglu et al. (2026) | Türkiye | Volleyball | 2 | 68; 34/34 | 15.95±1.43 | FR vs DS | FR; gastrocnemius, quadriceps, hamstrings, and gluteals; bilateral 1 × 30 s per muscle group. DS; same muscle groups; bilateral dynamic stretching. | 0 min | ② | CMJ |
| Faigenbaum et al. (2006) | USA | NR | 2 | 30; NR | 15.5±0.9 | SS vs DS vs SS-DS | SS; five static stretching exercises; 2 × 30 s  DS; nine dynamic exercises; 2 × 10 yd  SS-DS; 5 min of SS followed by 5 min of DS; SS 1 × 30 s + DS 1 × 10 yd | 1–2 min | ②, ④ | VJ; 10 yd sprint |
| Flórez-Gil et al. (2025) | Spain, Portugal | Basketball | 3 | 15; 0/15 | 16.7±0.6 | WU vs PAPE vs DS vs HEAT | PAPE; single-leg low-hurdle step-over, continuous multiple bounds, and single-leg vertical jump; repeated cycles of 2 min rest and 1 min activity  DS; high knees, butt kicks, forward/lateral lunges, and leg swings; repeated cycles of 2 min rest and 1 min activity  HEAT; towel wrapping of the head and lower limbs; seated passive heat maintenance for 15 min | 0 min | ②, ④ | CMJ; 505 test |
| Gergley (2010) | USA | Golf | 2 | 9; 9/0 | 20.4±1.8 | DS vs SS-DS | DS; 10 swings with a 1.13 kg weighted bar + 15 dynamic swings progressing from short iron to fairway wood + 3 maximal-effort fairway-wood swings; 1 min between repetitions  SS-DS; 20 min whole-body passive static stretching (12 exercises, 3 × 10 s) + the same DS protocol | 0 min | ① | Hitting distance |
| Gibson et al. (2013) | UK | Team sports: football, field hockey, and rugby | 4 | 25; 16/9 | 22.9±3.2 | WU vs SHAM vs IPC | IPC; alternating bilateral legs; 3 × (5 + 5 min); 220 mmHg | 5 min | ④ | 10 m sprint time |
| Gibson et al. (2015) | UK | Team sports: football, field hockey, and rugby | 4 | 16; 7/9 | 24.1±2.6 | WU vs SHAM vs IPC | IPC; alternating bilateral legs; 3 × (5 + 5 min); 220 mmHg | 5 min | ② | Peak power |
| Gonçalves et al. (2013) | Brazil | Football | 2 | 18; 18/0 | 15.78±0.81 | SS vs DS | SS; four lower-limb exercises; static hold  DS; four lower-limb exercises; 10 s hold + 5 s isometric contraction; repeated 4 times | 0 min | ④ | 50 m sprint velocity |
| Hill et al. (2024) | UK | Swimming | 2 | 24; 13/11 | 16.7±1.2 | WU vs PAPE | PAPE; medicine-ball slam (5 kg for females, 9 kg for males); 3 × 5 repetitions; 2 min between sets | 8 min | ① | 50 m freestyle swimming time |
| Hittinger et al. (2015) | USA | Cycling, triathlon | 2 | 15; 15/0 | 29.9±6.6 | WU vs IPC | IPC; bilateral legs; 4 × (5 + 5 min); SBP + 10–20 mmHg | 45 min | ② | Peak power |
| Işık and Şenel (2023) | Türkiye | Football | 2 | 10; 10/0 | 15.8±1.47 | WU vs SS vs DS | SS; eight static stretching exercises; approximately 15 s per exercise; 5–10 s rest between exercises  DS; 15 dynamic exercises; total duration 10 min | 1–2 min | ②, ④ | Medicine-ball throwing distance; T-test agility |
| Kamiş et al. (2025) | Türkiye | Handball | 3 | 12; 12/0 | 20.08±3.12 | IPC vs SHAM | IPC; bilateral legs; 3 × (2 + 2 min); 100% AOP | 10 min | ②, ④ | SJ; Zigzag test |
| Kanik et al. (2025) | Türkiye | Canoeing | 2 | 13; 13/0 | 13.07±1.32 | DS vs FR vs DS-FR | DS; 12 dynamic exercises; 2 × 15 s per exercise  FR; nine muscle groups; 2 × 20 s per muscle group  DS-FR; FR followed by DS | 0 min | ② | VJ |
| Kilding et al. (2018) | New Zealand | Cycling | 2 | 8; 8/0 | 27.0±7.0 | IPC vs SHAM | IPC; bilateral legs; 4 × (5 + 5 min); 200 mmHg | 5 min | ③ | Peak aerobic power |
| Kilduff et al. (2011) | UK | Swimming | 4 | 9; 7/2 | 22.0±2.0 | PAPE vs WU | PAPE; back squat; 1 × 3 repetitions; 87% 1RM. | 8 min | ① | 15 m swim-start time |
| Kilduff et al. (2013b) | UK | Rugby | 2 | 20; 20/0 | 26.0±2.0 | WU vs HEAT | HEAT; insulated jacket; 15 min | 0 min | ②, ④ | CMJ peak power output; 6 × 40 m sprint time |
| Kourtis-Doulkeridis et al. (2025) | Greece | Basketball | 2 | 15; 15/0 | 13.4±0.5 | SS vs DS | SS; static stretching; 3 × 10 s; 10 s between sets; 30 s between exercises  DS; slow dynamic stretching; 3 × 10 s; 50 bpm | 1 min | ②, ④ | CMJ; 10 m sprint time |
| Krčmár et al. (2015) | Slovakia | Ice hockey, volleyball, rowing, karate | 2 | 11; NR | 22.0±1.8 | WU vs PAPE | PAPE; back half-squat; 2 × 4RM; knee angle 90°; 3 min between sets | 10 min | ② | Maximal mean power output |
| Kurt and Fırtın (2016) | Türkiye | Football | 2 | 20; 20/0 | 25.3±4.3 | WU vs SS vs DS | SS; six unilateral exercises, 1 × 20 s each, with 10 s between exercises + one bilateral exercise, 2 × 20 s  DS; seven dynamic exercises; 2 × 20 s; 10 s between exercises | 0 min | ②, ④ | Relative peak power; Illinois agility test |
| Kurt et al. (2023) | Türkiye | Handball, basketball | 2 | 23; 0/23 | 21.8±1.73 | SS vs DS vs FR | SS; three exercises; bilateral 3 × 30 s; 30 s between exercises  DS; eight exercises; bilateral 2 × 20 s; 15 s between sets; 30 s between exercises  FR; bilateral; 3 × 30 s; 15 s between sets; 30 s between exercises | 2 min | ② | CMJ |
| Kurt et al. (2024) | Türkiye | Wrestling | 2 | 17; 17/0 | 20.0±4.06 | WU vs DS | DS; seven dynamic exercises; 2 × 10 repetitions; 100 bpm; 10 s between sets; 30 s between exercises | 2 min | ② | Relative power |
| Langdown et al. (2019) | UK | Golf | 2 | 23; 22/1 | 19.4–19.7 | WU vs PAPE vs DS | PAPE; five resistance-band exercises; 10 min  DS; five exercises; 10 min | 0 min | ① | Ball flight distance |
| Lima et al. (2011) | Brazil | Athletics | 4 | 10; 10/0 | 20.6±2.6 | WU vs PAPE | PAPE; depth jump; 2 × 5 repetitions; 0.75 m; 15 s between jumps; 3 min between sets | 5 min | ②, ④ | CMJ; 50 m sprint time |
| Lisbôa et al. (2017) | Brazil | Swimming | 2 | 11; 11/0 | 20.0±3.0 | IPC vs SHAM | IPC; bilateral legs at 220 mmHg + bilateral upper limbs at 180 mmHg; 4 × (5 + 5 min) | 60 min | ① | 50 m swimming sprint time |
| Liu R. D. et al. (2024) | China | Sprinting | 3 | 24; 24/0 | 22.2±2.2 | WU vs PAPE | PAPE; depth jump; 5 repetitions; 45 cm; unloaded | 8 min | ④ | 20 m sprint time |
| Lopez-Samanes et al. (2021) | Spain | Tennis | 4 | 11; 11/0 | 20.6±3.5 | DS vs FR | DS; 8 min dynamic warm-up; 3 sets with progressive intensity; 15 s between sets  FR; foam rolling; 60 s each; total duration 8 min | 0 min | ②, ④ | CMJ; 10 m sprint time |
| Loughran et al. (2017) | UK | Gaelic football | 4 | 17; 17/0 | 20.9 | WU vs SS vs SS-DS | SS; five exercises; 30 s each  SS-DS; SS followed by DS; SS: five exercises, 30 s each + DS: ten exercises, 2 × 30 s | 10 min | ②, ④ | CMJ; 40 m sprint time |
| Low et al. (2015) | Canada | Football | 2 | 16; 16/0 | 17.05±0.65 | WU vs PAPE | PAPE; barbell squat; 3 sets; 91% 1 RM | 8 min | ④ | 6 × 35 m sprint time |
| Low et al. (2019) | Canada | Middle- and long-distance running | 2 | 12; 12/0 | 28.33±1.37 | WU vs PAPE | PAPE; elastic-band resisted squat jump; 4 × 5RM; 2 min between sets | 8 min | ③ | 5 km time |
| Lowery et al. (2014) | USA | Distance running | 3 | 10; 10/0 | 24.0±5.0 | SS vs WU | SS; six lower-limb stretching exercises; 3 × 30 s per exercise; 30 s between sets. | 3 min | ③ | 1-mile uphill (5%) run completion time |
| Ltifi et al. (2023) | Tunisia | Football | 2 | 12; 12/0 | 16.3±0.3 | WU vs SS vs DS vs SS-DS vs DS-SS | SS; six static stretching exercises; total duration 6 min  DS; ten dynamic exercises; total duration 6 min  SS-DS; SS followed by DS; total duration 12 min  DS-SS; DS followed by SS; total duration 12 min | 2 min | ④ | Illinois agility test |
| Lum (2019) | Singapore | Judo | 4 | 11; 11/0 | 16–29 | WU vs PAPE | PAPE; standing long jump; 2 × 5 repetitions; 1 min between sets | 5 min | ① | Total number of throws in the Special Judo Fitness Test |
| Mascarin et al. (2015) | Brazil | Handball | 4 | 21; 0/21 | 16.2±1.0 | WU vs SS vs SS-DS | SS; five shoulder exercises; 3 × 30 s; 15 s between sets  SS-DS; SS followed by DS; elastic-band dynamic shoulder warm-up; two exercises, 10 repetitions each | 0 min | ①, ② | Handball throwing velocity; medicine-ball throwing distance |
| Matthews et al. (2009) | UK | Mixed sports | 2 | 12; 12/0 | 21.8±4.5 | WU vs PAPE | PAPE; bench press; 1 × 5 repetitions; 85% 1RM | 4 min | ① | Basketball push-pass flight time |
| Matthews et al. (2010) | UK | Ice hockey | 2 | 11; NR | 22.09±3.05 | WU vs PAPE | PAPE; resisted on-ice sprint; 1 × 10 s | 4 min | ① | 25 m on-ice sprint time |
| McGowan et al. (2016) | Australia | Swimming | 4 | 10; 6/4 | 20.4±2.07 | WU vs HEAT | HEAT; heated pants; 51 °C | 0 min | ① | 100 m breaststroke time |
| McGowan et al. (2017) | Australia | Swimming | 4 | 25; 12/13 | 20.0±2.48 | WU vs HEAT | HEAT; heated pants; 51 °C | 0 min | ① | 100 m freestyle time |
| Mersin and Agopyan (2025) | Türkiye | Swimming | 2 | 19; 0/19 | 11.07±1.42 | WU vs FR | FR; vibrating foam rolling; 4 × 30 s; 10 s between sets. | 0 min | ① | 25 m kick time |
| Mojock et al. (2011) | USA | Long-distance running | 2 | 12; 0/12 | 30.0±9.0 | WU vs SS | SS; five exercises; 4 repetitions each; total duration 18 min | 0 min | ③ | Endurance running distance |
| Munro et al. (2017) | New Zealand | Track cycling | 4 | 6; 4/2 | 19.2±3.2 | WU vs PAPE | PAPE; cycle ergometer dynamic activation; 4 × 4 crank cycles; 2 min between sets | 4 min | ②, ④ | Single short-distance sprint power; time |
| Nelson et al. (2023), Trial 1 | USA | Cycling | 2 | 11; 9/2 | 26.8±17.6 | IPC vs SHAM | IPC; alternating bilateral legs; 3 × (5 + 5 min); 100% occlusion | 20 min | ③ | Mean power output during a 3 min time trial |
| Nelson et al. (2023), Trial 2 | USA | Cycling | 2 | 13; 9/4 | 23.93±6.25 | IPC vs SHAM | IPC; alternating bilateral legs; 3 × (5 + 5 min); 100% occlusion | 20 min | ③ | Mean power output during a 6 min time trial |
| O’Brien et al. (2026) | Canada | Mixed sports: kayaking, rowing, dragon boating, weightlifting, and martial arts | 3 | 18; 9/9 | 23.0±4.0 | IPC vs SHAM | IPC; bilateral upper arms; 4 × (5 min occlusion + 5 min reperfusion); individual LOP determined by loss of distal pulse. | 42 min | ② | 45-s arm-crank WAnT absolute mean power output |
| Ormeno and Driller (2025) | Australia | Basketball, football, Australian rules football, and netball | 2 | 16; 12/4 | 23.2±1.3 | DS vs DS-FR | DS; Dynamic warm-up; eight exercises. DS-FR; dynamic warm-up + bilateral foam rolling of the quadriceps, hamstrings, and calves; 2 min per site; total duration 12 min. | 3 min | ②, ④ | CMJ; 20 m sprint time |
| Ou et al. (2026) | China | Taekwondo | 2 | 16; 16/0 | 19.25±1.29 | IPC vs SHAM | IPC; alternating bilateral legs; 4 × (5 + 5 min); 220 mmHg | 20 min | ① | Total number of kicks across three sets of the FSKT |
| Petisco et al. (2019) | Spain | Football | 2 | 10; 10/0 | 21.6±3.2 | WU vs PAPE | PAPE; half-squat; 1 × 5 repetitions; 80% 1RM | 6 min | ②, ④ | CMJ; COD |
| Poulos et al. (2018) | Tunisia | Volleyball | 4 | 15; 15/0 | 24.3±2.6 | WU vs PAPE | PAPE; back squat; 3 repetitions per set; 87% 1RM | 6 min | ② | CMJ |
| Rêma et al. (2024) | Portugal | Athletics | 2 | 20; 11/9 | 21.0 | WU vs SS | SS; quadriceps, hamstrings, gastrocnemius, and tibialis anterior; 3 min per muscle group bilaterally | 0 min | ② | Long jump performance |
| Ribeiro et al. (2019) | Brazil | Judo | 4 | 12; 12/0 | 22.0±3.69 | IPC vs SHAM | IPC; bilateral legs; 3 × (5 + 5 min); 220 mmHg | 30 min | ① | Special Judo Fitness Test index |
| Richard and Billaut (2018) | Canada | Athletics | 4 | 9; 7/2 | 23.3±2.6 | RIPC vs SHAM | RIPC; bilateral upper limbs; 3 × (5 + 5 min); SBP + 30 mmHg | 60 min | ③ | 1000 m time-trial time |
| Romero-Franco et al. (2019) | Spain | Mixed sports | 2 | 30; 18/12 | 24.6±4.4 | FR vs WU | FR; posterior thigh, anterior thigh, and calf; bilateral 1 × 45 s per muscle group; 15 s between legs; total duration ≈6 min. | 0 min | ② | CMJ |
| Rumeau et al. (2023) | France | Ice hockey | 3 | 11; 0/11 | 17.3±1.1 | WU vs PAPE | PAPE; maximal concentric leg press; 1 × 15 s | 0 min | ②, ④ | CMJ; 20 m sprint time |
| Sanchez-Sanchez et al. (2018) | Spain | Football | 3 | 16; NR | 20.75±1.21 | WU vs PAPE | PAPE; explosive squat; 1 set; approximately 90% 1RM; 0.5 m/s | 5 min | ④ | Fastest 20 m sprint time |
| Sánchez-Sánchez et al. (2025) | Spain | High jump | 2 | 20; NR | 20.8±0.5 | WU vs PAPE | PAPE; loaded squat; 1 set; target velocity 0.8 m/s; 5% velocity loss | 4 min | ②, ④ | CMJ; 5 m sprint time |
| Santos et al. (2026) | Brazil | Brazilian jiu-jitsu | 3 | 17; 17/0 | 33.8±6.2 | IPC vs SHAM | IPC; alternating proximal bilateral thighs; 3 × (5 + 5 min); 220 mmHg; total duration 30 min | 5 min | ①, ② | Total number of repetitions in the JJAPT; CMJ |
| Sayers et al. (2008) | USA | Football | 4 | 20; 0/20 | 19.35±0.99 | WU vs SS | SS; static stretching of the hamstrings, calves, and quadriceps; 3 × 30 s per leg | 0 min | ④ | 30 m sprint time |
| Seitz et al. (2014) | Australia, France | Rugby | 4 | 13; NR | 18.8±0.9 | WU vs PAPE | PAPE; power clean; 1 × 3 repetitions; 90% 1RM | 7 min | ④ | 20 m sprint time |
| Taghidoust Chahardeh et al. (2026) | Iran | Taekwondo | 3 | 36; 0/36 | 21.5±2.0 | FR vs DS | FR; gluteals, hamstrings, quadriceps, and gastrocnemius; 3 × 30 s per muscle group; 15 s between sets. DS; eight dynamic exercises; 2 × 20 s per exercise; 15 s between sets. | 0 min | ② | DJ |
| Turnes et al. (2018) | Brazil | Rowing | 3 | 16; 16/0 | 24.0±11.0 | IPC vs SHAM | IPC; proximal thigh; 3 × (5 + 5 min); 220 mmHg | 45 min | ③ | 2000 m rowing time |
| West et al. (2016) | UK, France | Rugby | 2 | 15; 15/0 | 28.0±3.0 | WU vs PAPE vs HEAT | HEAT; insulated jacket; 20 min  PAPE; loaded countermovement jump (CMJ); 3 × 5 repetitions; 20% body mass | 8 min | ④ | Fastest time in the 6 × 40 m shuttle sprint |
| Wilson et al. (2010) | USA | Middle- and long-distance running | 2 | 10; 10/0 | 25.0±7.0 | WU vs SS | SS; knee flexors, plantar flexors, quadriceps, hip flexors, and gluteus maximus; 4 × 30 s per exercise; total duration 16 min | 0 min | ③ | Distance covered during a 30 min endurance run |
| Yang et al. (2022) | China | Snowboarding | 2 | 16; 16/0 | 16.72±1.15 | WU vs FR | FR; triceps surae, tibialis anterior, quadriceps, adductors, tensor fasciae latae, hamstrings, and gluteus maximus; 30 s per side; total duration 7 min | 0 min | ②, ④ | SJ; 30 m sprint time |
| Yang et al. (2023) | China | Racket sports: table tennis, badminton, and tennis | 2 | 24; 12/12 | 22.2±2.0 | WU vs SHAM vs RIPC | RIPC; bilateral upper limbs; 5 × (5 + 5 min); 220 mmHg | 30 min | ③ | time to exhaustion during a supramaximal test |
| Yang et al. (2024) | China | Rhythmic gymnastics | 4 | 20; 0/20 | 18.25±2.55 | WU vs PAPE | PAPE; squat jump; 6 repetitions; individualized load of 6.9 W/kg; 15 s between repetitions | 9 min | ② | CMJ |
| Zanni et al. (2026) | Greece | Athletics | 2 | 14; 9/5 | 16.86±1.70 | PAPE vs WU | PAPE; bilateral horizontal drop jump; 1 × 5 repetitions; individualized optimal drop height; 10 s between repetitions. | 4 min | ④ | 30 m sprint time |
| Zinner et al. (2017) | Germany, Switzerland | Team sports: football, basketball, and handball | 2 | 13; 7/6 | 24.0±2.0 | IPC vs RIPC vs SHAM | IPC; bilateral legs; 3 × (5 + 5 min); 220 mmHg  RIPC; bilateral upper limbs; 3 × (5 + 5 min); 190 mmHg | 45 min | ④ | Mean sprint time over 16 × 30 m |
| Zisi et al. (2022) | Greece | Sprinting | 2 | 12; 5/7 | 17.2±1.6 | WU vs PAPE | PAPE; resisted sled towing; 2 × 20 m; individualized load; 2 min between sets | 8 min | ④ | 30 m sprint time |

Note: N, total sample size; M/F, male/female; NR, not reported; WU, regular warm-up; PAPE, post-activation performance enhancement; IPC, ischemic preconditioning; RIPC, remote ischemic preconditioning; SHAM, sham stimulation; SS, static stretching; DS, dynamic stretching; SS-DS, static stretching followed by dynamic stretching; DS-SS, dynamic stretching followed by static stretching; FR, foam rolling; HEAT, passive heat maintenance; DS-FR, dynamic stretching combined with foam rolling; CMJ, countermovement jump; SJ, squat jump; VJ, vertical jump; COD, change-of-direction ability; PSTT, sport-specific time-to-exhaustion test; IOP, individual occlusion pressure; SBP, systolic blood pressure. AOP, arterial occlusion pressure; bpm, beats per minute; DJ, drop jump; FSKT, frequency speed of kick test; JJAPT, jiu-jitsu anaerobic performance test; LOP, limb occlusion pressure; MVIC, maximal voluntary isometric contraction; RM, repetition maximum; WAnT, Wingate anaerobic test. Outcome categories were coded as follows: ① sport-specific performance; ② explosive/jump performance; ③ endurance performance; ④ speed/change-of-direction performance.

References

Aandahl, H. S., von Heimburg, E., and van den Tillaar, R. (2018). Effect of postactivation potentiation induced by elastic resistance on kinematics and performance in a roundhouse kick of trained martial arts practitioners. J. Strength Cond. Res. 32, 990–996. doi: 10.1519/JSC.0000000000001947

Albuquerque, M. R., Flôr, C. A. G., Ribeiro, A. I. S., Mesquita, P. H. C., Franchini, E., and Laurentino, G. C. (2024). Effects of ischemic preconditioning on sport-specific performance in highly trained taekwondo athletes. Sports (Basel) 12, 179. doi: 10.3390/sports12070179

Andrić, N., Jezdimirović-Stojanović, T., Mikić, M., Međedović, B., Jakšić, D., and Stojanović, M. D. M. (2026). Acute effects of flywheel eccentric overload on vertical jump, change of direction, and isometric mid-thigh pull performance in top-level team sports athletes. Sports (Basel) 14, 6. doi: 10.3390/sports14010006

Aydın, S., Eken, Ö., and Aldhahi, M. I. (2026). Acute effects of squat loading intensity on performance in competitive judo athletes. Sci. Rep. 16, 7819. doi: 10.1038/s41598-026-39312-x

Aytac, T., Esatbeyoglu, F., and Kin-Isler, A. (2024). Post-activation performance enhancement on change of direction speed: effects of heavy back-squat exercise. Sci. Sports 39, 196–205. doi: 10.1016/j.scispo.2023.04.005

Baiget, E., Colomar, J., and Corbi, F. (2023). Joint-specific postactivation potentiation enhances serve velocity in young tennis players. J. Strength Cond. Res. 37, 840–847. doi: 10.1519/JSC.0000000000004334

Barbosa, T. M., Yam, J. W., Lum, D., Balasekaran, G., and Marinho, D. A. (2020). Arm-pull thrust in human swimming and the effect of post-activation potentiation. Sci. Rep. 10, 8464. doi: 10.1038/s41598-020-65494-z

Bayazit, B., and Turna, B. (2022). Post-activation potentiation (PAP): effect on target performance in archery. Int. J. Life. Sci. Pharma Res. 11, 71–75.

Beaven, C. M., Kilduff, L. P., and Cook, C. J. (2018). Lower-limb passive heat maintenance combined with pre-cooling improves repeated sprint ability. Front. Physiol. 9, 1064. doi: 10.3389/fphys.2018.01064

Blagrove, R. C., Holding, K. M., Patterson, S. D., Howatson, G., and Hayes, P. R. (2019). Efficacy of depth jumps to elicit a post-activation performance enhancement in junior endurance runners. J. Sci. Med. Sport 22, 239–244. doi: 10.1016/j.jsams.2018.07.023

Brink, N. J., Constantinou, D., and Torres, G. (2022a). Postactivation performance enhancement (PAPE) of sprint acceleration performance. Eur. J. Sport Sci. 22, 1411–1417. doi: 10.1080/17461391.2021.1955012

Brink, N. J., Constantinou, D., and Torres, G. (2022b). Postactivation performance enhancement (PAPE) using a vertical jump to improve vertical jump performance. J. Sports Med. Phys. Fitness 62, 1419–1426. doi: 10.23736/S0022-4707.22.12899-9

Çabuk, S., and İnce, İ. (2025). The acute effects of hip thrust and glute bridge exercises with different loads on sprint performance and horizontal force–velocity profile in adolescent soccer players: a post-activation performance enhancement approach. Eur. J. Sport Sci. 25, e12255. doi: 10.1002/ejsc.12255

Ceylan, B., and Franchini, E. (2022). Ischemic preconditioning does not improve judo-specific performance but leads to better recovery in elite judo athletes. Sci. Sports 37, 322.e1–322.e7. doi: 10.1016/j.scispo.2021.08.004

Chaouachi, A., Castagna, C., Chtara, M., Brughelli, M., Turki, O., Galy, O., et al. (2010). Effect of warm-ups involving static or dynamic stretching on agility, sprinting, and jumping performance in trained individuals. J. Strength Cond. Res. 24, 2001–2011. doi: 10.1519/JSC.0b013e3181aeb181

Chen, Y., Yang, J., Muradov, O., Li, X., Lee, J. K. W., and Qiu, J. (2023). Effect of ischemic preconditioning on maximum accumulated oxygen deficit in 400-meter runners. Eur. J. Sport Sci. 23, 789–796. doi: 10.1080/17461391.2022.2064769

Chua, M. T., Chow, K. M., Lum, D., Tay, A. W. H., Goh, W. X., Ihsan, M., et al. (2021). Effectiveness of on-court resistive warm-ups on change of direction speed and smash velocity during a simulated badminton match play in well-trained players. J. Funct. Morphol. Kinesiol. 6, 81. doi: 10.3390/jfmk6040081

Cocking, S., Wilson, M. G., Nichols, D., Cable, N. T., Green, D. J., Thijssen, D. H. J., et al. (2018). Is there an optimal ischemic-preconditioning dose to improve cycling performance? Int. J. Sports Physiol. Perform. 13, 274–282. doi: 10.1123/ijspp.2017-0114

Cuenca-Fernández, F., Konrad, A., and Tilp, M. (2026). Stretching and potentiation for performance optimization: effects on upper limbs in competitive swimmers. Eur. J. Appl. Physiol. 126, 3071–3086. doi: 10.1007/s00421-026-06133-9

de Oliveira, F. C. L., and Rama, L. M. P. L. (2016). Static stretching does not reduce variability, jump and speed performance. Int. J. Sports Phys. Ther. 11, 237–246.

de Paula Simola, R.Á., Almeida, P. A. S., da Silva, S. F., Rabelo, A. S., and Gonçalves, R. (2026). Acute effect of mechanical vibration on sprint performance in mountain bike athletes. Int. J. Perform. Anal. Sport 26, 857–868. doi: 10.1080/24748668.2025.2578106

Dello Iacono, A., Padulo, J., Eliakim, A., Gottlieb, R., Bareli, R., and Meckel, Y. (2016). Post-activation potentiation effects on vertical and horizontal explosive performances of young handball and basketball athletes. J. Sports Med. Phys. Fitness 56, 1455–1464.

Di Cagno, A., Baldari, C., Battaglia, C., Gallotta, M. C., Videira, M., Piazza, M., et al. (2010). Preexercise static stretching effect on leaping performance in elite rhythmic gymnasts. J. Strength Cond. Res. 24, 1995–2000. doi: 10.1519/JSC.0b013e3181e34811

dos Santos Silva, D., Ferreira, A. R. P., Aidar, F. J., Gama de Matos, D., Kalinine, E., da Silva-Grigoletto, M. E., et al. (2021). Post-activation potentiation effect after acute plyometric exercises on the performance of long jumpers. Muscles Ligaments Tendons J. 11, 494–499. doi: 10.32098/mltj.03.2021.14

Eyuboglu, F., Ayhan, Y., and Kolaylı Çerezci, T. (2026). Acute effects of fascial applications on performance and flexibility in adolescent volleyball athletes. J. Bodyw. Mov. Ther. 47, 406–411. doi: 10.1016/j.jbmt.2026.04.015

Faigenbaum, A. D., Kang, J., McFarland, J., Bloom, J. M., Magnatta, J., Ratamess, N. A., et al. (2006). Acute effects of different warm-up protocols on anaerobic performance in teenage athletes. Pediatr. Exerc. Sci. 18, 64–75. doi: 10.1123/pes.18.1.64

Flórez-Gil, E., Mateus, N., Sampaio, J., and Abade, E. (2025). Examining the impact of different re-warm-up strategies on non-starter basketball players’ physical performance. J. Sports Sci. 43, 536–543. doi: 10.1080/02640414.2025.2467564

Gergley, J. C. (2010). Latent effect of passive static stretching on driver clubhead speed, distance, accuracy, and consistent ball contact in young male competitive golfers. J. Strength Cond. Res. 24, 3326–3333. doi: 10.1519/JSC.0b013e3181e725e4

Gibson, N., White, J., Neish, M., and Murray, A. (2013). Effect of ischemic preconditioning on land-based sprinting in team-sport athletes. Int. J. Sports Physiol. Perform. 8, 671–676. doi: 10.1123/ijspp.8.6.671

Gibson, N., Mahony, B., Tracey, C., Fawkner, S., and Murray, A. (2015). Effect of ischemic preconditioning on repeated sprint ability in team sport athletes. J. Sports Sci. 33, 1182–1188. doi: 10.1080/02640414.2014.988741

Gonçalves, D. L., Pavão, T. S., and Dohnert, M. B. (2013). Acute and chronic effects of a static and dynamic stretching program in the performance of young soccer athletes. Rev. Bras. Med. Esporte 19, 241–246. doi: 10.1590/S1517-86922013000400003

Hill, V., Patterson, S., Buckthorpe, M., and Legg, H. S. (2024). The acute effects of a preload upper-body power exercise on 50-m freestyle performance in youth swimmers. J. Strength Cond. Res. 38, 1295–1299. doi: 10.1519/JSC.0000000000004792

Hittinger, E. A., Maher, J. L., Nash, M. S., Perry, A. C., Signorile, J. F., Kressler, J., et al. (2015). Ischemic preconditioning does not improve peak exercise capacity at sea level or simulated high altitude in trained male cyclists. Appl. Physiol. Nutr. Metab. 40, 65–71. doi: 10.1139/apnm-2014-0080

Işık, M., and Şenel, Ö. (2023). The acute effects of different stretching exercises on the power and agility of adolescent football player. Int. J. Sport Cult. Sci. 11, 150–166. doi: 10.14486/IntJSCS.2023.683

Kamiş, O., Akçay, N., Keskin, K., Rolnick, N., García Sánchez, C., de Queiros, V. S., et al. (2025). Short-duration ischemic preconditioning does not improve jump or change of direction performance in elite male handball players. PloS One 20, e0334747. doi: 10.1371/journal.pone.0334747

Kanik, G., Kargın, Z., Karakaş, F., Keskin, H., and Mor, A. (2025). Acute effects of dynamic stretching and foam rolling warm-up protocols on certain performance parameters in canoeists. J. Sport All Recreat. 7, 427–437. doi: 10.56639/jsar.1698583

Kilding, A. E., Sequeira, G. M., and Wood, M. R. (2018). Effects of ischemic preconditioning on economy, VO2 kinetics and cycling performance in endurance athletes. Eur. J. Appl. Physiol. 118, 2541–2549. doi: 10.1007/s00421-018-3979-8

Kilduff, L. P., Cunningham, D. J., Owen, N. J., West, D. J., Bracken, R. M., and Cook, C. J. (2011). Effect of postactivation potentiation on swimming starts in international sprint swimmers. J. Strength Cond. Res. 25, 2418–2423. doi: 10.1519/JSC.0b013e318201bf7a

Kilduff, L. P., West, D. J., Williams, N., and Cook, C. J. (2013b). The influence of passive heat maintenance on lower body power output and repeated sprint performance in professional rugby league players. J. Sci. Med. Sport 16, 482–486. doi: 10.1016/j.jsams.2012.11.889

Kourtis-Doulkeridis, A., Koutsouridis, C., Galazoulas, C., and Karamousalidis, G. (2025). Acute effects of static and dynamic stretching on vertical jump and sprint performance in adolescent basketball players. J. Phys. Educ. Sport 25, 1036–1043. doi: 10.7752/jpes.2025.05114

Krčmár, M., Šimonek, J., and Vasiľovský, I. (2015). The acute effect of lower-body training on average power output measured by loaded half-squat jump exercise. Acta Gymnica 45, 103–111. doi: 10.5507/ag.2015.016

Kurt, C., and Fırtın, İ. (2016). Comparison of the acute effects of static and dynamic stretching exercises on flexibility, agility and anaerobic performance in professional football players. Turk. J. Phys. Med. Rehabil. 62, 206–213. doi: 10.5606/tftrd.2016.32698

Kurt, C., Gürol, B., and Özsu Nebioğlu, İ. (2023). Effects of traditional stretching versus self-myofascial release warm-up on physical performance in well-trained female athletes. J. Musculoskelet. Neuronal Interact. 23, 61–71.

Kurt, C., Tuna, G., and Kurtdere, İ. (2024). Acute effects of slow, moderate and fast tempo dynamic stretching exercises on power in well-trained male wrestlers. J. Hum. Kinet. 93, 155–165. doi: 10.5114/jhk/183543

Langdown, B. L., Wells, J. E. T., Graham, S., and Bridge, M. W. (2019). Acute effects of different warm-up protocols on highly skilled golfers’ drive performance. J. Sports Sci. 37, 656–664. doi: 10.1080/02640414.2018.1522699

Lima, J. C. B., Marin, D. P., Barquilha, G., da Silva, L. O., Puggina, E. F., Pithon-Curi, T. C., et al. (2011). Acute effects of drop jump potentiation protocol on sprint and countermovement vertical jump performance. Hum. Mov. 12, 324–330. doi: 10.2478/v10038-011-0036-4

Lisbôa, F. D., Turnes, T., Cruz, R. S. O., Raimundo, J. A. G., Pereira, G. S., and Caputo, F. (2017). The time dependence of the effect of ischemic preconditioning on successive sprint swimming performance. J. Sci. Med. Sport 20, 507–511. doi: 10.1016/j.jsams.2016.09.008

Liu, R. D., Zhang, J. X., Zeng, Z. C., Maiwulanjiang, T., and Zhang, J. J. (2024). The influence of post-activation potentiation based on drop jump exercises on the acceleration sprinting and muscle-tendon stiffness of elite male sprinters. J. Capital Univ. Phys. Educ. Sports 36, 561–569. doi: 10.14036/j.cnki.cn11-4513.2024.05.011

Lopez-Samanes, A., Del Coso, J., Hernández-Davó, J. L., Moreno-Pérez, D., Romero-Rodriguez, D., Madruga-Parera, M., et al. (2021). Acute effects of dynamic versus foam rolling warm-up strategies on physical performance in elite tennis players. Biol. Sport 38, 595–601. doi: 10.5114/biolsport.2021.101604

Loughran, M., Glasgow, P., Bleakley, C., and McVeigh, J. (2017). The effects of a combined static-dynamic stretching protocol on athletic performance in elite Gaelic footballers: a randomised controlled crossover trial. Phys. Ther. Sport 25, 47–54. doi: 10.1016/j.ptsp.2016.11.006

Low, D., Harsley, P., Shaw, M., and Peart, D. (2015). The effect of heavy resistance exercise on repeated sprint performance in youth athletes. J. Sports Sci. 33, 1028–1034. doi: 10.1080/02640414.2014.979857

Low, J. L., Ahmadi, H., Kelly, L. P., Willardson, J., Boullosa, D., and Behm, D. G. (2019). Prior band-resisted squat jumps improves running and neuromuscular performance in middle-distance runners. J. Sports Sci. Med. 18, 301–315.

Lowery, R. P., Joy, J. M., Brown, L. E., Oliveira de Souza, E., Wistocki, D. R., Davis, G. S., et al. (2014). Effects of static stretching on 1-mile uphill run performance. J. Strength Cond. Res. 28, 161–167. doi: 10.1519/JSC.0b013e3182956461

Ltifi, M. A., Jlid, M. C., Coquart, J., Maffulli, N., van den Tillaar, R., and Aouadi, R. (2023). Acute effect of four stretching protocols on change of direction in U-17 male soccer players. Sports (Basel) 11, 165. doi: 10.3390/sports11080165

Lum, D. (2019). Effects of various warm-up protocol on special judo fitness test performance. J. Strength Cond. Res. 33, 459–465. doi: 10.1519/JSC.0000000000001862

Mascarin, N. C., Vancini, R. L., Lira, C. A. B., and Andrade, M. S. (2015). Stretch-induced reductions in throwing performance are attenuated by warm-up before exercise. J. Strength Cond. Res. 29, 1393–1398. doi: 10.1519/JSC.0000000000000752

Matthews, M., O’Conchuir, C., and Comfort, P. (2009). The acute effects of heavy and light resistances on the flight time of a basketball push-pass during upper body complex training. J. Strength Cond. Res. 23, 1988–1995. doi: 10.1519/JSC.0b013e3181b3e076

Matthews, M. J., Comfort, P., and Crebin, R. (2010). Complex training in ice hockey: the effects of a heavy resisted sprint on subsequent ice-hockey sprint performance. J. Strength Cond. Res. 24, 2883–2887. doi: 10.1519/JSC.0b013e3181e7253c

McGowan, C. J., Pyne, D. B., Thompson, K. G., and Rattray, B. (2016). Evaluating warm-up strategies for elite sprint breaststroke swimming performance. Int. J. Sports Physiol. Perform. 11, 975–978. doi: 10.1123/ijspp.2015-0581

McGowan, C. J., Pyne, D. B., Thompson, K. G., Raglin, J. S., Osborne, M., and Rattray, B. (2017). Elite sprint swimming performance is enhanced by completion of additional warm-up activities. J. Sports Sci. 35, 1493–1499. doi: 10.1080/02640414.2016.1223329

Mersin, H. T., and Agopyan, A. (2025). Acute effects of traditional and self-myofascial release warm-up protocols on ankle flexibility and short-distance swimming performance in young competitive swimmers. J. Bodyw. Mov. Ther. 45, 886–893. doi: 10.1016/j.jbmt.2025.10.019

Mojock, C. D., Kim, J. S., Eccles, D. W., and Panton, L. B. (2011). The effects of static stretching on running economy and endurance performance in female distance runners during treadmill running. J. Strength Cond. Res. 25, 2170–2176. doi: 10.1519/JSC.0b013e3181e859db

Munro, L. A., Stannard, S. R., Fink, P. W., and Foskett, A. (2017). Potentiation of sprint cycling performance: the effects of a high-inertia ergometer warm-up. J. Sports Sci. 35, 1442–1450. doi: 10.1080/02640414.2016.1215492

Nelson, C. R., Brand, C. R., Chitty, M. R., Birger, C. B., and Scholten, S. D. (2023). The acute effects of ischemic preconditioning on short-duration cycling: a randomized crossover study. Int. J. Exerc. Sci. 16, 148–158. doi: 10.70252/EGXC5574

O’Brien, L., McLaughlin, M., Gillen, J., and Jacobs, I. (2026). The effects of ischemic preconditioning and placebo maneuvers are similar during arm-cranking performance in upper-body trained athletes. Eur. J. Appl. Physiol. 126, 3017–3029. doi: 10.1007/s00421-026-06197-7

Ormeno, L., and Driller, M. (2025). Does massage gun or foam roller use during a warm-up improve performance in trained athletes? Sports (Basel) 13, 282. doi: 10.3390/sports13090282

Ou, Z., Yang, L., Xie, D., Zhu, H., Weng, X., and Xu, G. (2026). Ischemic preconditioning enhances energy supply during frequency speed kick test in Taekwondo athletes: a randomized crossover study. PloS One 21, e0341780. doi: 10.1371/journal.pone.0341780

Petisco, C., Ramirez-Campillo, R., Hernández, D., Gonzalo-Skok, O., Nakamura, F. Y., and Sanchez-Sanchez, J. (2019). Post-activation potentiation: effects of different conditioning intensities on measures of physical fitness in male young professional soccer players. Front. Psychol. 10, 1167. doi: 10.3389/fpsyg.2019.01167

Poulos, N., Chaouachi, A., Buchheit, M., Slimani, D., Haff, G. G., and Newton, R. U. (2018). Complex training and countermovement jump performance across multiple sets: effect of back squat intensity. Kinesiology 50, 75–89.

Rêma, S., Seixas, A., Moreira-Silva, I., Cardoso, R., Ventura, N., and Azevedo, J. (2024). Influence of different durations of lower limb static stretching on the performance of long jump athletes: a randomized controlled trial. Sport Sci. Health 20, 73–78. doi: 10.1007/s11332-023-01066-8

Ribeiro, A. A. S., Novaes, J., Reis, N., Telles, L. G., Sant'Ana, L., Raider, L., et al. (2019). Acute effect of ischemic preconditioning on the performance and on the hemodynamic responses of high-performance male judo athletes. JEPonline 22, 154–164.

Richard, P., and Billaut, F. (2018). Time-trial performance in elite speed skaters after remote ischemic preconditioning. Int. J. Sports Physiol. Perform. 13, 1308–1316. doi: 10.1123/ijspp.2018-0111

Romero-Franco, N., Romero-Franco, J., and Jiménez-Reyes, P. (2019). Jogging and practical-duration foam-rolling exercises and range of motion, proprioception, and vertical jump in athletes. J. Athl. Train. 54, 1171–1178. doi: 10.4085/1062-6050-474-18

Rumeau, V., Grospretre, S., and Babault, N. (2023). Post-activation performance enhancement and motor imagery are efficient to emphasize the effects of a standardized warm-up on sprint-running performances. Sports (Basel) 11, 108. doi: 10.3390/sports11050108

Sanchez-Sanchez, J., Rodriguez, A., Petisco, C., Ramirez-Campillo, R., Martínez, C., and Nakamura, F. Y. (2018). Effects of different post-activation potentiation warm-ups on repeated sprint ability in soccer players from different competitive levels. J. Hum. Kinet. 61, 189–197. doi: 10.1515/hukin-2017-0131

Sánchez-Sánchez, J., Luis-Pereira, J. M., and Carretero-González, M. (2025). Impact of an activation protocol on jump and acceleration performance in high jump athletes. Cultura Cienc. Deporte 20, 2242. doi: 10.12800/ccd.v20i66.2242

Santos, I. A., da Silva, B. V. C., de Oliveira, D. C. X., da Cruz, G. G. C., Puggina, E. F., and Mota, G. R. (2026). Ischemic preconditioning slightly enhances jiu-jitsu performance. Int. J. Sports Med. 47, 295–303. doi: 10.1055/a-2722-8807

Sayers, A. L., Farley, R. S., Fuller, D. K., Jubenville, C. B., and Caputo, J. L. (2008). The effect of static stretching on phases of sprint performance in elite soccer players. J. Strength Cond. Res. 22, 1416–1421. doi: 10.1519/JSC.0b013e318181a450

Seitz, L. B., Trajano, G. S., and Haff, G. G. (2014). The back squat and the power clean: elicitation of different degrees of potentiation. Int. J. Sports Physiol. Perform. 9, 643–649. doi: 10.1123/ijspp.2013-0358

Taghidoust Chahardeh, S., Hosseini, S. H., and Shamsi Majelan, A. (2026). Acute effects of three warm-up protocols on drop jump biomechanics in elite Taekwondo athletes: an IMU-based analysis. PloS One 21, e0351884. doi: 10.1371/journal.pone.0351884

Turnes, T., de Aguiar, R. A., de Oliveira Cruz, R. S., and Caputo, F. (2018). Impact of ischaemia–reperfusion cycles during ischaemic preconditioning on 2000-m rowing ergometer performance. Eur. J. Appl. Physiol. 118, 1599–1607. doi: 10.1007/s00421-018-3891-2

West, D. J., Russell, M., Bracken, R. M., Cook, C. J., Giroud, T., and Kilduff, L. P. (2016). Post-warmup strategies to maintain body temperature and physical performance in

Wilson, J. M., Hornbuckle, L. M., Kim, J. S., Ugrinowitsch, C., Lee, S. R., Zourdos, M. C., et al. (2010). Effects of static stretching on energy cost and running endurance performance. J. Strength Cond. Res. 24, 2274–2279. doi: 10.1519/JSC.0b013e3181b22ad6

Yang, J., Li, W. H., Liu, J. J., Hu, Y., Yang, X. G., and Wang, J. R. (2022). The acute effects of self-fascial relaxation warm-up on snowboarders’ performance. Liaoning Sport Sci. Technol. 44, 75–81.

Yang, J. C., Yan, S. T., Chen, M. G., Chen, Y. Y., and Qiu, J. Q. (2023). Effects of remote ischemic preconditioning on maximal accumulated oxygen deficit in racquet athletes. Chin. J. Sports Med. 42, 772–779.

Yang, C., Shi, L., Lu, Y., Wu, H., and Yu, D. (2024). Post-activation performance enhancement of countermovement jump after drop jump versus squat jump exercises in elite rhythmic gymnasts. J. Sports Sci. Med. 23, 611–618. doi: 10.52082/jssm.2024.611

Zanni, E., Stavridis, I., Zacharogiannis, E., Chatzakis, P., Argeitaki, P., and Paradisis, G. (2026). Acute effect of bilateral horizontal drop jumps in sprint and jumping performance and sprint mechanical and kinematics characteristics. Biomechanics 6, 10. doi: 10.3390/biomechanics6010010

Zinner, C., Born, D. P., and Sperlich, B. (2017). Ischemic preconditioning does not alter performance in multidirectional high-intensity intermittent exercise. Front. Physiol. 8, 1029. doi: 10.3389/fphys.2017.01029

Zisi, M., Stavridis, I., Agilara, G. O., Economou, T., and Paradisis, G. (2022). The acute effects of heavy sled towing on acceleration performance and sprint mechanical and kinematic characteristics. Sports (Basel) 10, 77. doi: 10.3390/sports10050077
